# ImmunoPET Predicts Response to Met-targeted Radioligand Therapy in Models of Pancreatic Cancer Resistant to Met Kinase Inhibitors

**DOI:** 10.7150/thno.37098

**Published:** 2020-01-01

**Authors:** Freddy E. Escorcia, Jacob L. Houghton, Dalya Abdel-Atti, Patricia R. Pereira, Andrew Cho, Nicholas T. Gutsche, Kwamena E. Baidoo, Jason S. Lewis

**Affiliations:** 1 Department of Radiology, Memorial Sloan Kettering Cancer Center, 1275 York Avenue, New York, New York, 10065, USA.; 2 Department of Radiation Oncology, Memorial Sloan Kettering Cancer Center, 1275 York Avenue, New York, New York, 10065, USA.; 3 Molecular Imaging Program, Center for Cancer Research, National Cancer Institute, National Institutes of Health, Bethesda, MD 20814, USA.; 4 Department of Radiology and Radiological Sciences, Vanderbilt University Institute of Imaging Science, Nashville, TN 37232, USA.; 5 Department of Biochemistry & Structural Biology, Weill Cornell Graduate School, New York City, New York 10065, USA.; 6 Weill Cornell/Rockefeller/Sloan Kettering Tri-Institutional MD-PhD Program, New York City, New York 10065, USA.; 7 Program in Molecular Pharmacology, Memorial Sloan Kettering Cancer Center, 1275 York Avenue, New York, New York, 10065, USA.; 8 Weill Cornell Medical College, 1300 York Ave, New York, New York, 10065, USA.

**Keywords:** ImmunoPET, pancreatic cancer, molecular imaging.

## Abstract

**Background**: Pancreatic ductal adenocarcinoma (PDAC) has limited standard of care therapeutic options. While initially received with enthusiasm, results from targeted therapy with small molecule tyrosine kinases inhibitors (TKIs) have been mixed, in part due to poor patient selection and compensatory changes in signaling networks upon blockade of one or more kinase of tumors. Here, we demonstrate that in PDACs otherwise resistant to rational kinase inhibition, Met-directed immuno-positron emission tomography (immunoPET) can identify targets for cell-signaling independent targeted radioligand therapy (RLT). In this study, we use Met-directed immunoPET and RLT in models of human pancreatic cancer that are resistant to Met- and MEK-selective TKIs, despite over-expression of Met and KRAS-pathway activation.

**Methods**: We assessed cell membrane Met levels in human patient samples and pancreatic ductal adenocarcinoma (PDAC) cell lines (BxPC3, Capan2, Suit2, and MIA PaCa-2) using immunofluorescence, flow cytometry and cell-surface biotinylation assays. To determine whether Met expression levels correlate with sensitivity to Met inhibition by tyrosine kinase inhibitors (TKIs), we performed cell viability studies. A Met-directed imaging agent was engineered by labeling Met-specific onartuzumab with zirconium-89 (Zr-89) and its *in vivo* performance was evaluated in subcutaneous and orthotopic PDAC xenograft models. To assess whether the immunoPET agent would predict for targeted RLT response, onartuzumab was then labeled with lutetium (Lu-177) as the therapeutic radionuclide to generate our [^177^Lu]Lu-DTPA-onartuzumab RLT agent. [^177^Lu]Lu-DTPA-onartuzumab was administered at 9.25MBq (250μCi)/20μg in three fractions separated by three days in mice subcutaneously engrafted with BxPC3 (high cell-membrane Met) or MIA PaCa-2 (low cell-membrane Met). Primary endpoints were tumor response and overall survival.

**Results**: Flow cytometry and cell-surface biotinylation studies showed that cell-membrane Met was significantly more abundant in BxPC3, Capan2, and Suit2 when compared with MIA PaCa-2 pancreatic tumor cells. Crizotinib and cabozantinib, TKIs with known activity against Met and other kinases, decreased PDAC cell line viability *in vitro*. The TKI with the lowest IC_50_ for Met, capmatinib, had no activity in PDAC lines. No additive effect was detected on cell viability when Met-inhibition was combined with MEK1/2 inhibition. We observed selective tumor uptake of [^89^Zr]Zr-DFO-onartuzumab in mice subcutaneously and orthotopically engrafted with PDAC lines containing high cell-surface levels of Met (BxPC3, Capan2, Suit2), but not in mice engrafted with low cell-surface levels of Met (MIA PaCa-2). Significant tumor growth delay and overall survival benefit were observed in both BxPC3 and MIA PaCa-2 engrafted animals treated with RLT when compared to controls, however, the benefit was more pronounced and more durable in the BxPC3 engrafted animals treated with [^177^Lu]Lu-DTPA-onartuzumab RLT.

**Conclusions**: Our findings demonstrate that while over-expression of Met is not predictive of Met-directed TKI response, immunoPET can detect Met over-expression *in vivo* and predicts for therapeutic response to Met-selective RLT. This phenomenon can be exploited for other Met-overexpressing tumor types specifically, and to any differentially overexpressed surface molecule more broadly.

## Introduction

The standard of care for patients diagnosed with locally advanced pancreatic cancer is systemic chemotherapy. Still, only a minority (<25%) of patients achieve any radiographic response, and the 5-year survival for all patients is <5% 1, 2. While new approaches to delivering cytotoxic chemotherapy are being explored, the absolute overall survival remains poor in these predominantly Kirsten RAS (KRAS)-activated pancreatic cancer patients and new therapies are needed 3.

Activating mutations in the KRAS onocogene are present in >90% of pancreatic adenocarcinoma 4. In an attempt to alter the course of this fatal disease, targeted small molecule drugs have been investigated to complement cytotoxic chemotherapy. For example, KRAS inhibitors, as well as inhibitors of associated kinases have been developed, including RAF, the mitogen-activated protein kinase (MAPK), the extracellular signal-regulated kinase (ERK), and the MAPK/ERK kinase (MEK) 5, 6. Notably, recent studies have shown activating KRAS mutations may cause compensatory signaling changes that later abrogate the need for KRAS activation 7. Combined small molecule therapies have also been studied, including inhibition of receptor tyrosine kinases (RTKs), such as fibroblast growth factor receptor (FGFR), with promising synthetic lethality in lung cancer models, though with modest results in pancreatic cancer models 8. Furthermore, given the relatively low percentage of patients with “actionable” alterations in their cancers, trials to date have produced mixed results, underscoring the need for new treatment paradigms and strategies for selecting patients who are likely to respond to a given course of therapy 9, 10.

Identifying patients with potentially actionable somatic events that would lead to therapeutic response is difficult. While histopathological staining for tumor markers and genomic analysis all play a crucial role toward this end, neither accurately represent marker availability *in vivo*. For example, immunohistochemistry (IHC) may overestimate target accessibility because some stained targets are intracellular and unavailable to engage antibody-based therapies *in vivo*, resulting in a suboptimal selection of patients since antibodies usually target membrane domains of the antigen/receptors. Although TKIs can access intracellular targets, understanding whether their receptors are overexpressed, accessible, and contributing to oncogenesis *in vivo* may inform not only targeted therapy, but also treatment with biologic agents. A theranostic approach with molecular immunoPET can help begin answering some of these questions 11, 12.

The RTK hepatocyte growth factor (HGF) receptor, Met, is overexpressed in several malignancies, including cancers of the lung, breast, bladder, and pancreas 13, 14. While normal ductal cells rarely express Met, it is over-expressed in up to 80% of invasive of PDAC. Furthermore, Met over-expression is associated with poor overall patient survival, and increased recurrence rates following surgery 15, 16. Similarly, >90% of PDAC cell lines exhibit high expression of cell-membrane Met 16. Combined, these observations suggest that Met may be a useful therapeutic target in pancreatic cancer. Given reports of underwhelming responses to Met-inhibition in unselected populations, the value of patient selection cannot be overemphasized 9.

Because Met activates the KRAS pathway, we hypothesized that in cells that overexpress Met, combined blockade of the RAS pathway and Met would yield therapeutic synergy. This rationale was even specifically highlighted in previous reports on the interplay between Met signaling and KRAS mutant cancers 17. Our findings, however, disproved this hypothesis. Herein lies the challenge of precision oncology: despite identification of overexpressed or constitutive activation of certain molecules in cancer, blockade of associated molecular pathways may be insufficient to yield therapeutic benefit 18. A therapy that can exploit this over-expression independent of complex signaling is needed, and RLT is one option.

While Met expression is not predictive of response to Met targeted TKIs, we posit that detection and targeting of Met may have value as a theranostic tool to identify Met-expressing tumors that may respond to RLT. This opens an avenue in precision medicine where the “actionable mutation” biology (and evolution of resistance mutations/mechanisms) is less relevant as long as a molecular target can be identified and thus, therapeutically targeted.

The technology to engineer molecular imaging agents from biologic agents, peptides or small molecules in order to directly visualize both on-target and off-target localization in patients has been available for many years, but remains underutilized clinically, despite having the potential to provide insights into responses to therapy, including TKIs 19-23. Successes of molecular imaging targeting prostate specific membrane antigen (PSMA) and its role in guiding standard as well as new treatments with RLT agents, including alpha-particle emitters, serve as a model for how such agents could be deployed 24-27.

Onartuzumab is a one-armed humanized monoclonal antibody that binds to the extracellular domain of Met, blocking HGF binding, receptor activation, dimerization, internalization, and limiting degradation or shedding of Met 28. Onartuzumab also has a lower molecular weight (99 kDa) when compared with full-length antibodies (150 kDa), engendering it with comparatively faster blood clearance *in vivo* and yielding better signal to noise for imaging earlier following initial tracer injection. Accordingly, we used this antibody as a scaffold from which to build a Met selective immunoPET and RLT agent. To the best of our knowledge, no one has evaluated onartuzumab in KRAS-activated PDAC for either imaging or radiotherapeutic purposes 29-33.

## Materials and Methods

### Expression of Met in human cancers & in human pancreatic cancer cell lines

Previously published gene expression array datasets of tumors and matched normal tissue from patients were interrogated for differential expression of Met by combining tools from oncomine.org and evaluating original published data 34-41. Kaplan-Meier curves were generated using data from pancreatic adenocarcinoma patients (n = 177) from The Cancer Genome Atlas (TCGA). Clinical data were downloaded from the National Institutes of Health Genomic Data Commons Data Portal, and Met mRNA levels from pancreatic adenocarcinoma patients were downloaded from cBioPortal (www.cbioportal.org). The datasets were parsed with an in-house MATLAB script to stratify the patient population into two groups (n = 44) containing patients exhibiting Met expression levels in the top or bottom 25-percentile of the entire patient cohort. Survival time for each patient was extracted from the clinical data set, and Kaplan-Meier curves were generated for each group. To explore expression levels of Met in human cancer cell lines, we obtained Affymetrix HG-U133 chip-based microarray data from the Broad-Novartis Cancer Cell Line Encyclopedia (CCLE) website containing Robust Multi-array Average (RMA) normalized mRNA expression data with Affymetrix annotations across 1457 human cancer cell lines 42. The data were parsed with an in-house MATLAB script to extract upstream (MET, HGF) and downstream (MAPK1, MAPK3, AKT1, AKT2, and AKT3) mRNA expression values across 44 different human pancreatic cancer cell lines, and values annotated as 'Absent' were omitted. For genes with multiple reads on the Affymetrix Chip (MET, HGF, MAPK1, AKT2, and AKT3), the geometric mean of all expression values for a given gene was computed and used for subsequent calculations. Z-scores were calculated for ease of comparison across the genes of interest.

### Cell Lines and Patient Samples

PDAC (BxPC3, Suit2, Capan2, and MIA PaCa-2) cell lines were purchased from ATCC (Manassas, VA) and were grown according to standard procedures. All media were purchased from the Media Preparation Facility at Memorial Sloan Kettering Cancer Center (MSKCC). All cell lines were mycoplasma free and maintained at 37 °C in a humidified atmosphere at 5% CO_2_. Cell lines were authenticated at MSKCC integrated genomics operation core using short tandem repeat analysis, and used within passage number 15. Deidentified patient non-tumor and pancreatic cancer samples were obtained from David M. Rubenstein Center for Pancreatic Cancer Research following institutional review board (IRB) approval.

### Flow cytometry

Onartuzumab was produced and provided by Genentech (San Francisco, CA). Approximately 10 × 10^6^ cells of each PDAC line were harvested, and washed with ice-cold phosphate buffered saline (PBS) three times. Cell pellets were resuspended in FcR block (1:5 final dilution) (Miltenyi Biotec, Bergisch Gladbach, Germany) and incubated for 30 minutes on ice. Cell suspensions were then split into multiple groups, stained with fluorescently labeled onartuzumab (3µg/mL), fluorescently-labeled non-specific IgG (3µg/mL), or incubated without antibody for 30 minutes on ice. Following incubation, cells were washed with ice cold buffer (PBS, 2% FCS, 0.1% sodium azide, 1 mM ethylenediaminetetraacetic acid (EDTA)) three times 34. DAPI (Sigma-Aldrich, St. Louis, MO) was added prior to assaying samples. Single color controls were made and results were analyzed with FlowJo software (FlowJo LLC, Ashland, Oregon).

### Biotin Pull Down of Cell-Surface Proteins

For biotin pull-down assays, cells were washed twice with ice-cold PBS buffer containing 0.5 mM magnesium chloride (MgCl_2_) and 1 mM calcium chloride (CaCl_2_). Cells were incubated with 0.5 mg/mL of EZ-LINK Sulfo-Biotin (Thermo Fisher Scientific) for 30 min at 4 °C with gentle rotation. The reaction was stopped by washing twice with 100 mM glycine (Thermo Fisher Scientific) in PBS containing 0.5 mM MgCl_2_ and 1 mM CaCl_2_. Cells were scraped in RIPA buffer [RIPA buffer: 150 mM sodium chloride (NaCl), 50 mM Tris hydrochloride (Tris-HCl), pH 7.5, 5 mM ethylene glycol tetraacetic acid (EGTA), 1% Triton X-100, 0.5% sodium deoxycholate (DOC), 0.1% sodium dodecyl sulfate (SDS), 2 mM phenylmethanesulfonyl fluoride (PMSF), 2 mM iodoacetamide (IAD), and 1X protease inhibitor cocktail (Roche)], lysates were centrifuged at 16,000 *g* for 10 min at 4°C, and supernatants were collected and assayed for protein concentration using the Pierce BCA Protein Assay Kit (Thermo Fisher Scientific, Waltham, MA, USA). A volume of 500 μL of RIPA buffer containing equal amount of proteins was incubated with NeutrAvidin Agarose Resins (Thermo Fisher Scientific) for 2 h at 4 °C with gentle rotation and washed three times with RIPA buffer before suspension in Laemmli buffer.

### Western Blot Analysis

Whole-protein extracts from cells were prepared after cell scraping in RIPA assay buffer, centrifugation, protein quantification, and denaturation in Laemmli buffer as described above. Cell-membrane protein extracts were prepared as previously described. Following electrophoresis and transfer to polyvinylidene difluoride (PVDF) membranes (Bio-Rad, Hercules, CA), the blots were incubated in 5% (m/v) BSA in Tris-buffered saline buffer-Tween (TBS-T, Cell Signaling Technology, Danvers, MA) and probed with mouse anti β-actin 1:20,000 (Sigma-Aldrich, St. Louis, MO) and rabbit anti-Met 1:1000 (Abcam, Cambridge, MA). After washing, the membranes were incubated with IRDye^®^800CW anti-Rabbit or anti-Mouse IgG 1:15,000 (LI-COR Biosciences, Lincoln, NE) and imaged on the Odyssey Infrared Imaging System (LI-COR Biosciences) followed by densitometric analysis.

### Radiolabeling onartuzumab

#### Zr-89

Preparation of Zr-89 labeled onartuzumab ([^89^Zr]Zr-DFO-onartuzumab) was achieved in accordance with previously described methods, including conjugation of *p*-SCN-Bn-DFO (Macrocyclics, Plano, TX), purification, and subsequent radiolabeling 30, 43-47. Briefly, Onartzumab stock buffer was exchanged with gel filtration columns into PBS (Sephadex G-25, PD10 desalting column; GE Healthcare, Chicago, IL) and pH adjusted to 8.5 with 1 M Na_2_CO_3_. *p*-SCN-Bn-DFO was added in a 6:1 ratio and incubated at 37 ºC for 90 minutes followed by purification via PD-10 column into PBS (pH 7.4). Then, Zr-89 oxalate in oxalic acid (1 M) was neutralized to pH 7.0-7.2, using Na_2_CO_3_ (1 M) followed by addition of the appropriate construct in PBS (pH 7.4). The mixture was incubated at room temperature for 60 minutes and monitored using radio-iTLC with silica-gel impregnated glass-microfiber paper strips (iTLC-SG, Varian, Lake Forest, CA), using a mobile phase of aqueous solution of EDTA (50 mM, pH 5.5), and analyzed using an AR-2000 (Bioscan Inc., Washington, DC). Upon satisfactory radiolabeling, the reaction was quenched by addition of the same EDTA solution (10-20 μL) and the labeled construct was purified using gel-filtration chromatography with 0.9% saline. Radiochemical purity was assessed by radio-iTLC as described above. The Zr-89 used was produced at MSKCC via the ^89^Y(*p*,*n*)^89^Zr transmutation reaction on a TR19/9 variable-beam energy cyclotron (Ebco Industries, Richmond, British Columbia, Canada) 48.

#### Lu-177

Onartzumab stock buffer was exchanged with PD10 column into PBS and pH adjusted to 8.5 with 1 M Na_2_CO_3_. The bifunctional chelate, *p*-SCN-Bn-CHX-A"-DTPA (Macrocyclics, Plano, TX), resuspended in DMSO, and reacted with onartuzumab at a 10:1 ratio and incubated at 37ºC for 90 minutes. The construct was purified with PD10 column using 200 mM ammonium acetate (pH=5.5) containing 6 mg/mL ascorbic acid buffer for radiolabeling. Lu-177 that was obtained from ITM Isotope Technologies (Garching, Germany) and was incubated with purified onartuzumab-CHX-A'' conjugate at 37ºC for 60 minutes. Labeling was monitored using radio-iTLC with silica-gel impregnated glass-microfiber paper strips (iTLC-SG, Varian, Lake Forest, CA, analyzed using an AR-2000, Bioscan Inc., Washington, DC), eluted with a mobile phase of aqueous solution of EDTA (50 mM, pH 5.5). The product was purified using PD10 column equilibrated with 6 mg/mL ascorbic acid in PBS (pH=7) adjusted with 1M Na_2_CO_3_. Radiochemical purity was assessed by radio-iTLC as described above.

Details of characterization of the bioconjugates and radioconjugates including mass spectrometry analysis, serum stability, and pharmacokinetic profiles are described fully in the **Supplemental Information**.

### Immunoreactivity Measurements

The immunoreactivity of the [^89^Zr]Zr-DFO-onartuzumab and [^177^Lu]Lu-DTPA-onartuzumab was determined using BxPC3, a human pancreatic cancer cell line which highly expresses Met, via a previously reported Lindmo method 49, 50. Linear regression analysis of the background-corrected data was performed by plotting the ratio of the total to bound (total/bound) radioactivity against the inverse of the normalized cell concentration (1/normalized cell concentration).

### Cell internalization assays

For the internalization assays with [^89^Zr]Zr-DFO-onartuzumab, cells were incubated with cell culture medium in the presence of 30 μg/mL (303 nM) [^89^Zr]Zr-DFO-onartuzumab for a total period of 8 h at 37 °C. For the fractionated experiments, 10 μg/mL [^89^Zr]Zr-DFO-onartuzumab was added every 3 h for a total of 8 h and extracts were collected at 3 h, 6 h and 8 h post-incubation. Control experiments were also performed by incubating the cells with unconjugated Zr-89, including both a fractionated dose (3 × 0.018 MBq/well of Zr-89) and single dose (0.054 MBq/well. Media containing non-cell-bound radiotracer was removed and the cells were washed twice with PBS. Cell surface-bound radiotracer was collected by cells incubated at 4 °C for 5 min in 0.2 M glycine buffer containing 0.15 M NaCl, 4 M urea at pH 2.5. Internalized fraction was obtained after cell lysis with 1 M sodium hydroxide (NaOH). All samples were measured on a gamma counter calibrated for Zr-89.

### Murine subcutaneous & orthotopic xenograft models

All animal studies were conducted under IACUC approved protocols and in accordance with the Guide for the Care and Use of Laboratory Animals. Female athymic homozygous nude mice, strain Crl:NU(NCr)-Foxn1^nu^ (Charles River Laboratories, Wilmington, MA), age between 6-8 weeks, were subcutaneously xenografted with 5 × 10^6^ BxPC3, Capan 2, Suit 2, MIA PaCa-2 cells, suspended in 150 μL of a solution containing a 1:1 (v/v) mixture of Matrigel (Corning, Corning, NY) and cell suspension. Tumors were grown to a size of approximately 150-200 mm^3^ post implantation before imaging.

To better recreate tumor microenvironment barriers (e.g. collagen matrix) commonly observed in *de novo* pancreatic cancers, we also evaluated our probe localization in the orthotopic xenograft models using BxPC3, Capan 2, Suit 2, MIA PaCa-2 cells as previously described 51.

### PET imaging

For experiments with the subcutaneous xenograft model, mice (n = 5) were administered either 25-fold mass excess of unlabeled onartuzumab 48 h prior to injection of [^89^Zr]Zr-DFO-onartuzumab [1.8-2.7 MBq (50-72 μCi), 15μg ] (blocking group), or only [^89^Zr]Zr-DFO-onartuzumab [1.8-2.7 MBq (50-72 μCi), 15μg] via the lateral tail vein. Static scans (n=2) were recorded at the 24 h, 48 h, 72 h, and 120 h with a minimum of 12 million coincident events (8-25 min total scan time). Images were recorded on a microPET Focus scanner (Concorde Microsystems, Knoxville, Tennessee) as previously described 44, 45. All of the resulting images were analyzed using ASIPro VM software.

### PET/CT

Orthotopically engrafted mice (n = 3) were administered [^89^Zr]Zr-DFO-onartuzumab [1.8-2.7 MBq (50-72 μCi), 15 μg], via tail vein injection and images were acquired at 24 h, and 120 h. Static scans were recorded on an Inveon PET/CT scanner (Siemens Healthcare Global) at the various time points with a minimum of 30 million coincident events (10-30 min total scan time). Data sorting, reconstruction, and normalization were performed as previously reported 44, 45. Combined PET/CT images were processed using Inveon Research Workplace software and optimized to show localization of the PET signal.

### Biodistribution studies [^89^Zr]Zr-DFO-onartuzumab in BxPC3, and [^177^Lu]Lu-DTPA-onartuzumab

Biodistribution of [^89^Zr]Zr-DFO-onartuzumab was assessed in nu/nu athymic mice subcutaneously engrafted with BxPC3 tumors. Tumor volumes were measured with a Peira TM900 (Peira Scientific Instruments, Belgium) tumor measuring device and the mice were separated into five groups (n=4-5) with similar mean and median tumor volumes before being injected via the lateral tail vein (2.1±0.01 MBq, 56.0±1.6 μCi/15μg). Biodistribution studies were performed at 1, 24, 48, 72 and 120 h post-injection. In one cohort, blocking was achieved by injecting 25-fold excess mass of unlabeled onartuzumab 48 h prior to [^89^Zr]Zr-DFO-onartuzumab injection.

Biodistribution studies of [^177^Lu]Lu-DTPA-onartuzumab were performed in nu/nu athymic mice subcutaneously engrafted with BxPC3 and MIA PaCa-2. Animals were separated in to three groups (n=5) with similar tumor volumes and received [^177^Lu]Lu-DTPA-onartuzumab via tail vein (3.72±0.14 MBq, 100.6±3.9 μCi/15μg). Biodistribution studies were performed at 24, 48, and 120 h post-injection.

For both constructs, thirteen tissues including the tumor were collected. The mass of each organ was determined and the radioactivity of each sample was measured using a Wizard^2^ automatic gamma counter that was calibrated for the corresponding radioisotope, Zr-89 or Lu-177. A calibration curve that was generated from standards of known activity was used to convert counts into activity. The counts from each sample were decay and background corrected from the time of injection and the activity in each sample was converted to % ID/g by normalization to the total activity injected into the respective animal.

### Autoradiography, Immunohistochemistry, and Immunofluorescence

Tumors of animals utilized for the biodistribution studies above were harvested, embedded in Tissue-Plus Optimal Cutting Temperature (OCT) compound (Scigen, Gardena, CA), and frozen on dry ice. Tissues were cut in a series of 10-μm sections. Autoradiography was performed as previously described 44. Following autoradiography, sequential sections were submitted to the Molecular Cytology Core Facility at MSKCC for automated Met, and Ki-67 immunohistochemistry. Image analysis was performed with ImageJ (https://imagej.nih.gov/ij/) 52. For immunofluorescence, fixation of normal and PDAC tissues was performed using acetone/methanol fixation buffer. The slides were then rehydrated in PBS for 10 min and blocked in 10% goat serum (Thermo Fisher Scientific) for 30 min at room temperature. The tissues were incubated overnight in a humidified box with anti-Met primary antibody (Abcam, Cambridge, MA, USA) 1:100, v:v in PBS containing 0.02% BSA). The slides were washed three times with PBS and then incubated with secondary antibody (Alexa Fluor 488-conjugated goat anti-Rb 1:300, Thermo Fisher Scientific, Waltham, MA, USA) for 1 h at room temperature. Cell nucleus staining was then performed using DAPI and the slides were washed and mounted in glycergel (Agilent Technologies, Santa Clara, CA, USA). Anti-human 1-10 ug/mL Alexa Fluor 488 was used for binding to onartuzumab antibody.

### Radioimmunotherapy with [^177^Lu]Lu-DTPA-onartuzumab

Mice were subcutaneously engrafted with either BxPC3 (high Met expressing) or MIA PaCa-2 (low Met expressing) and tumors allowed to grow to ~150mm^3^ prior to randomization into groups (n=10). Groups were treated with (1) saline, (2) [^177^Lu]Lu-DTPA-onartuzumab 9.25 MBq(250μCi)/20 μg × 3 fractions (every three days), (3) 20μg onartuzumab × 3 (every three days). Animal tumor size, weight, and survival were monitored and animals were sacrificed if tumor volumes exceded 2000 mm^3^, or if they lost >20% body weight.

### Statistical Analysis

Quantitations of results were performed with Prism software (Version 7.0, GraphPad software, La Jolla, CA). An unpaired, two-tailed Student's t-test was used to analyze the data. Survival analysis determined with Mantel-Cox log-rank test. In all cases, a 95% confidence level (P < 0.05) was considered to represent a statistical difference in the data. Graphically, p<0.05 = *, p<0.01= **, p<0.0005 = ***, p<0.0001 = ****, NS: not significant, p >0.05).

## Results

### Met is overexpressed in several cancer types, including pancreatic cancers

**Figure 1:**
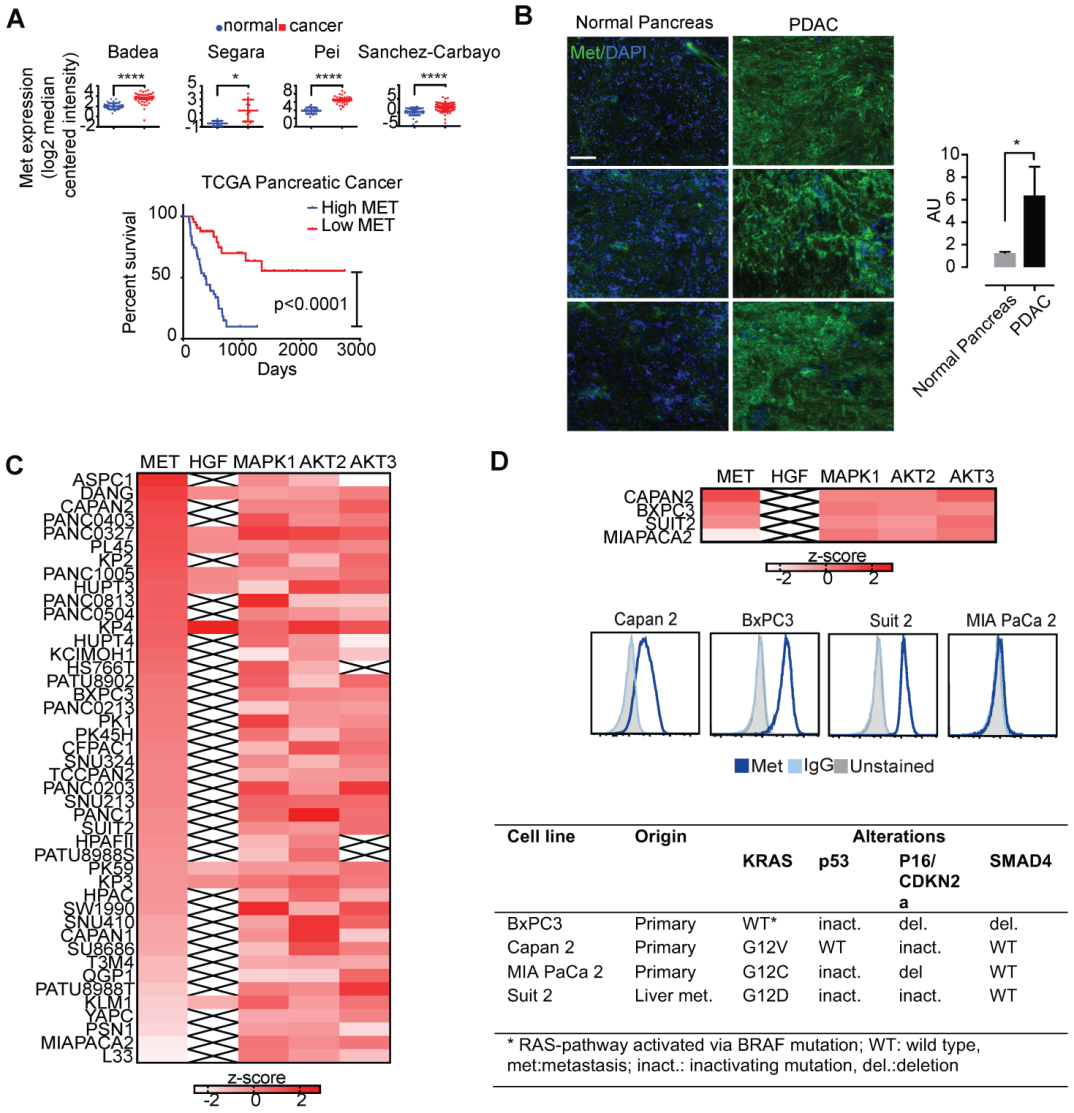
** Several human cancers demonstrate over-expression of Met. A.** Analysis of previously published patient gene expression datasets using oncomine.org listing log2 median centered intensity values across several (note: first author of relevant manuscript used to denote individual study, N=normal tissue, C=cancer). Kaplan-Meier survival curve of TCGA pancreatic cancer patients with high Met (top 25%) and low Met (bottom 25%) expression (Log-rank (Mantel-Cox) test, p <0.0001). **B.** patient matched normal pancreas (left) and pancreatic adenocarcinoma (right), and quantitation (ratio of green:blue, scale bar: 50μm). AU=arbitrary units is a ratio determined by measuring total of pixels positive for green (Met staining) versus total stain for blue (DAPI, cell nuclei). **C.** Heatmap denoting differential gene-expression of Met, HGF and downstream kinases for all pancreatic cancer cell lines in The Cancer Cell Line Encyclopedia, and, **D. (top panel)** focused heatmap of the cell lines used in the current study (“x”= null value on dataset). **D (middle panel)** Flow cytometry histograms confirming higher cell-membrane expression of Met in Capan 2, Suit 2, and BxPC3 when compared with MIA PaCa-2. **D (bottom panel)** Mutations present in four PDAC cell lines used in this study (note: while BxPC3 is RAS wildtype, it harbors an activating BRAF mutation). Error bars denote standard deviation (± s.d), p<0.05 = *, p<0.01= **, p<0.0005 = ***, p<0.0001 = ****, NS: not significant, p >0.05).

To determine Met expression in tumor vs. normal tissues, we first explored Met expression in a panel of tumor tissues (**Figure 1**). Using the cancer microarray database Oncomine across matched tumor and normal gene expression datasets, we confirmed amplification of Met in several pancreatic cancer patient datasets (denoted by the first author of the relevant publication) (**Figure 1A, top**). Analysis of pancreatic cancer data from The Cancer Genome Atlas (TCGA) demonstrates that Met expression is associated with unfavorable survival in patients with pancreatic adenocarcinoma (**Figure 1A, bottom**). Immunofluorescence studies in PDAC primary patient samples demonstrated higher expression in tumor tissue when compared with matched normal pancreas tissue (**Figure 1B**).

### KRAS-driven pancreatic cancer cells overexpress Met and are resistant to small molecule inhibition

Gene expression data from the cancer cell line encyclopedia (CCLE) assessing global Met expression along with its ligand, HGF, and other downstream kinases (MAPK1, AKT2, and AKT3) supported high Met expression in pancreatic cancer cell lines (**Figure 1C**). Together, these findings suggest the importance of Met and its downstream targets in pancreatic cancer. BxPC3, Capan2, and Suit2 pancreatic cancer cells exhibited high Met expression (**Figure 1B, D**). In contrast, MIA PaCa-2 pancreatic cancer cells demonstrated low expression of cell-membrane Met (**Figure 1C, D**). Results from flow cytometry with fluorescently-labeled onartuzumab (**Figure 1D**) incubated with the BxPC3, Capan2, Suit2, and MIA PaCa-2 PDAC cell lines were consistent with the expression data from CCLE (**Figure 1C, D**). Notably, these four cell lines exhibit mutational status common in PDAC including KRAS, TP53, p16/CDKN2A, SMAD4 (**Figure 1D**). While BxPC3 is KRAS wildtype, it harbors an activating BRAF mutation, making it functionally KRAS pathway-activated.

Given that PDAC overexpresses Met, we sought to determine the sensitivity of pancreatic cancer cells to Met-directed kinase inhibition. PDAC cell lines exhibit micromolar sensitivity to kinase inhibitors with activity to several kinases (crizotinib, cabozantinib), but are refractory to Met-specific inhibition (capmatinib/INC280) (**Table 1, S1**). A moderate effect on cell viability was observed when PDAC cell lines were exposed to MEK1/2 inhibition with trametinib; however, no additive cytotoxic effect was observed when trametinib was combined with capmatinib/INC280 (**Figure S1D**).

### Met-directed immunoPET identifies Met-expressing human pancreatic cancer *in vivo*

**Figure 2:**
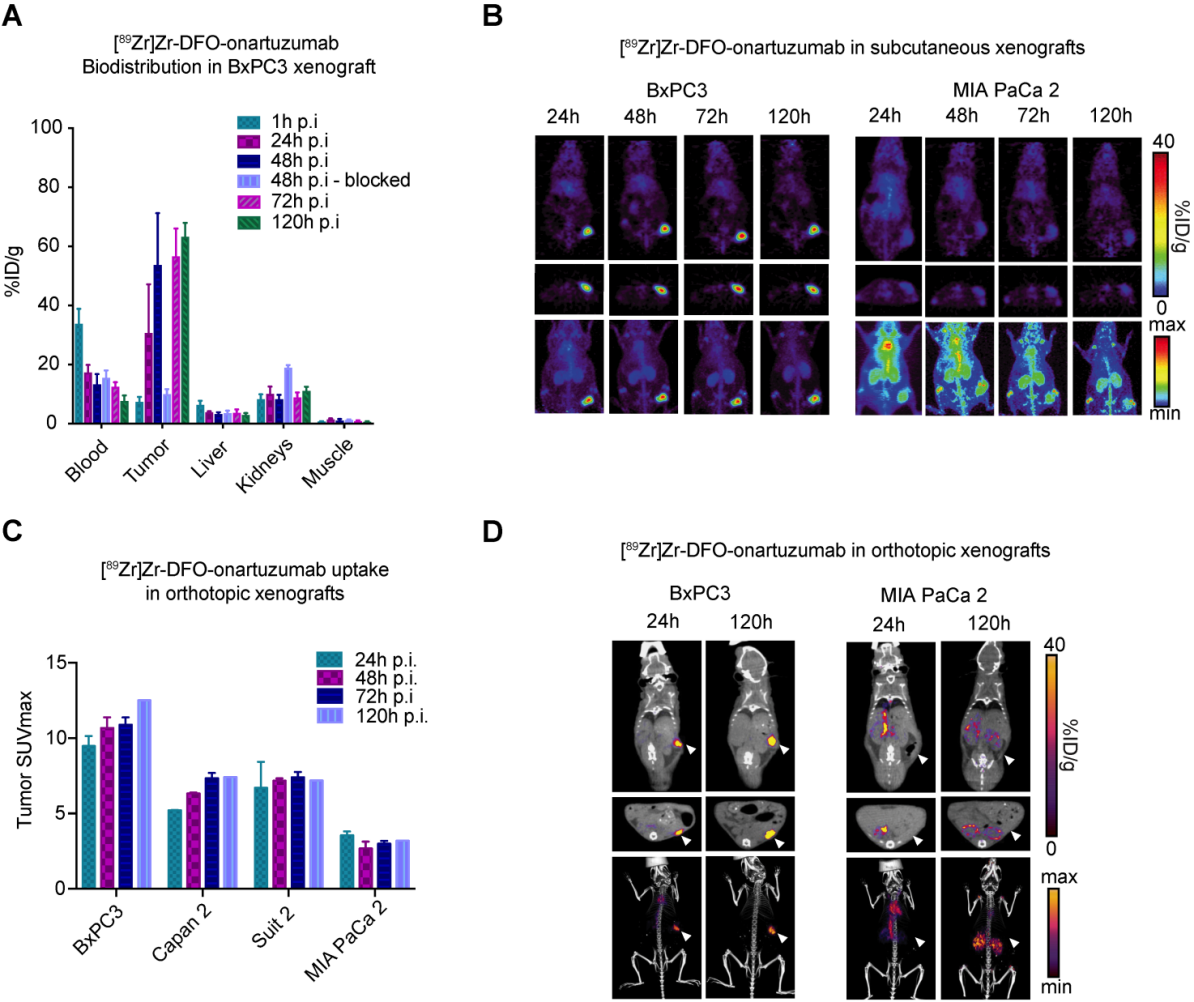
** [^89^Zr]Zr-DFO-onartuzumab accumulates in tumors expressing Met *in vivo*. A.** Quantitative biodistribution of [^89^Zr]Zr-DFO-onartuzumab in mice subcutaneously engrafted with BxPC3 at varying time points post injection (p.i.) denoted as percent injected dose per gram (%ID/g). **B.** Representative coronal (top), axial slices (bottom), and maximum intensity projection (MIP) PET images mice subcutaneously engrafted with BxPC3 (Met high) and MIA PaCa 2 (Met low) and evaluated at 24, 48, 72, and 120 h post injection. **C.** Quantitation of PET derived tumor SUVmax images of human pancreatic cancer cell lines subcutaneously engrafted into mice evaluated at a times post injection (n=2 for each timepoint except at 120h where n=1): BxPC3 (24h: 9.48±0.66, 48h: 10.68±0.70, 72h: 10.89±0.49, 120h: 12.52), Capan2 (24h: 5.22±0.01, 48h: 6.33±0.06, 72h: 7.34±0.36, 120h: 7.42), and Suit2 (24h: 6.71±1.72, 48h: 7.18±0.15, 72h: 7.40±0.35, 120h: 7.19), when compared to MIA PaCa-2 (24h: 3.56±0.25, 48h: 2.69±0.45, 72h: 2.99±0.19, 120h: 3.2). **D.** Representative PET images of human pancreatic cancer cell lines orthotopically engrafted into mice evaluated at 24, and 120h post injection; coronal (top), axial (middle), MIP bottom. Error bars denote standard deviation (± s.d), p<0.05 = *, p<0.01= **, p<0.0005 = ***, p<0.0001 = ****, NS: not significant, p >0.05).

Analysis of bioconjugates demonstrated 4.4 DFO and 0.44 DTPA per onartuzumab (**Figure S2A**), while serum stability and blood half-life studies suggested that our radioconugates would perform well *in vivo* (**Figure S2B,C**). To determine the ability of immunoPET to image Met-overexpressing pancreatic cancers, non-invasive imaging studies were performed in preclinical models of PDAC using [^89^Zr]Zr-DFO-onartuzumab. BxPC3 and MIA PaCa-2 exhibited the highest and lowest cell membrane Met levels, respectively (**Figure 1C**), and were therefore used for subsequent *in vivo* experiments. ^89^Zr-labeled onartuzumab allowed non-invasive tumor imaging in mice subcutaneously engrafted with BxPC3 **(Figure 2A)**. Notably, kidney accumulation was seen, which was comparable to prior studies with [^89^Zr]Zr-DFO-onartuzumab in other models, with corresponding increase in the group where Met was blocked with an excess of unlabeled antibody (8.45±1.3 versus 19.0±0.8 %ID/g, *p*=0.0004, n=4) (**Figure 2A, S3A,B**) 30, 33. PET imaging and biodistribution studies confirmed *in vivo* target engagement in subcutaneously engrafted models. PET images with high contrast were obtained as early as 24h after injection of ^89^Zr-labeled onartuzumab in BxPC3 tumors, and correspondingly low accumulation was observed in MIA PaCa-2 tumors as predicted (**Figure 2B**). PET SUVmax quantitation across all time points corresponded with *in vitro* Met expression with higher signal obtained in BxPC3 (**Figure 2C, S3B**). To recapitulate some components of the microenvironment in human pancreatic cancer, we evaluated our tracer in an orthotopic setting and similar performance was observed (**Figure 2D, S3C**). Biodistribution studies of [^89^Zr]Zr-DFO-onartuzumab were also performed with varied mass of the antibody to gauge the optimal mass needed to administer before saturating available receptors at the tumor and, therefore, decreasing the tumor uptake and degrading the signal-to-noise. We determined that < 25 μg of antibody would be optimal (**Figure S3A**).

### Fractionated dosing of radioconjugate enriches for cell membrane available Met

**Figure 3:**
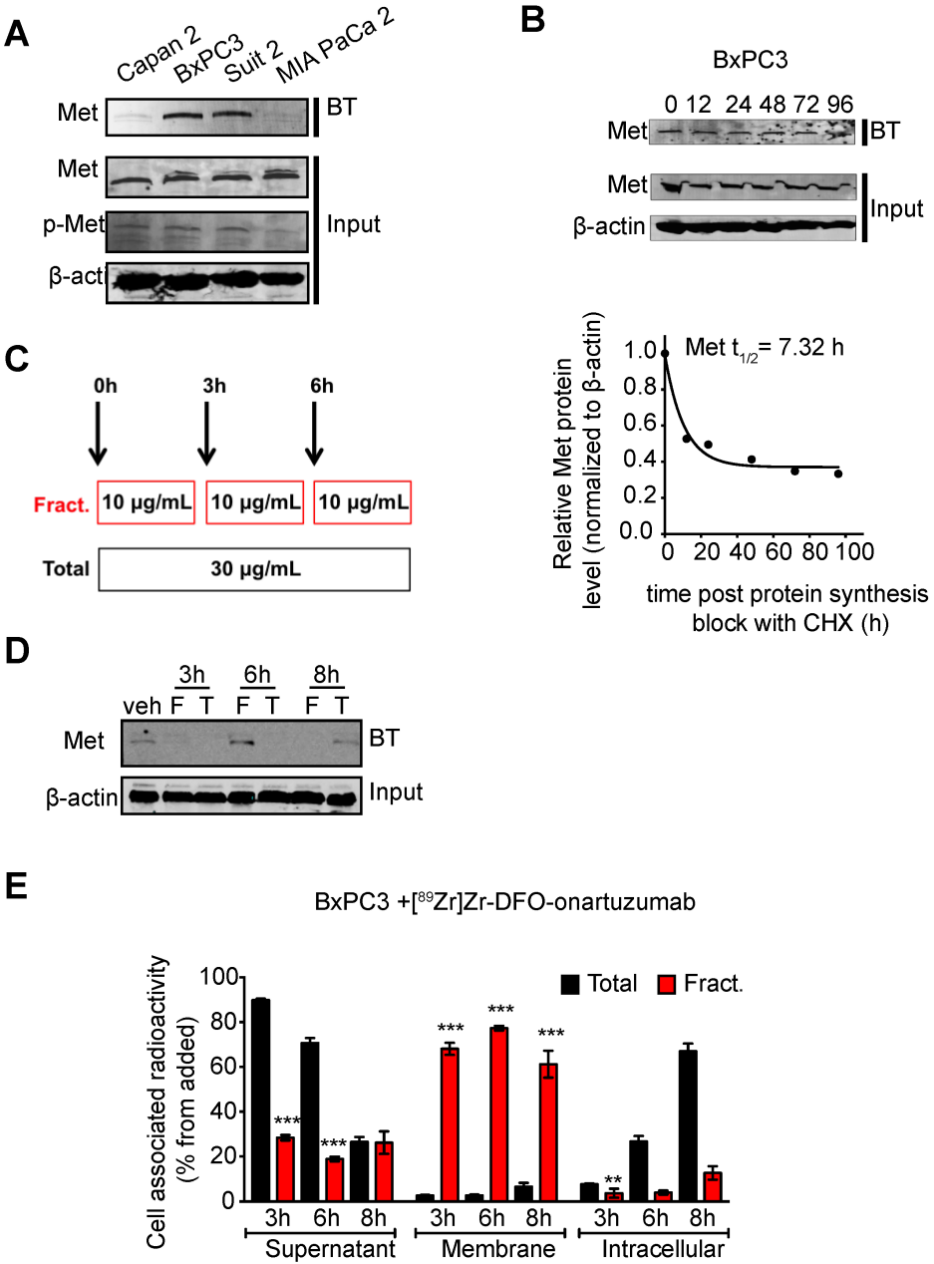
** Cell-membrane Met increases with fractionated treatment with onartuzumab. A**.Western blot of biotinylated cell surface-associated Met along with Met and phosphor-Met input in the total lysates of Capan 2, BxPC3, Suit2 and MIA PaCa2 pancreatic cancer cells. BT, biotinylation. **B.** Western blot of biotinylated cell surface-associated Met along with Met input in the total lysates of BxPC3 after blocking protein synthesis with 80 μg/mL CHX for 0, 12, 24, 48, 72 and 96h. CHX, cyclohexamide. Half-life of cell surface-associated Met calculated after western blot analysis. Density of western blot bands was quantified by scanning densitometry with ImageJ software. Half-life was calculated as the time required for Met protein decrease to 50% of its initial level. **C**. Schematic representation of the experimental protocol using a total or a fractionated dose of onartuzumab. Total dose (Total)- cells are incubated for 6 h with 30 μg/mL onartuzumab. Fractionated dose (Fract.) - cells are incubated 3-times with 10 μg/mL onartuzumab every 3h. **D**. Western blot of biotinylated cell surface-associated Met along with β-actin in BxPC3 cancer cells after cells incubation with a total or a fractionated dose of onartuzumab. Cells were incubated with onartuzumab as illustrated in Fig. 2c and cell extracts were prepared at 3, 6 and 8 h post treatment. Veh= vehicle, F=fractionated, T=total. **E.** Cellular localization of [^89^Zr]Zr-DFO-onartuzumab in BxPC3 cells. BxPC3 cells were incubated with a total or a fractionated dose of [^89^Zr]Zr-DFO-onartuzumab as illustrated in Figure 2C and radioactivity was measured at 3, 6 and 8 h post treatment in supernatant, membrane and intracellular fractions.

Encouraged by our previous studies demonstrating that membrane dynamics need to be considered in imaging and therapy of membrane receptors, we performed *in vitro* studies interrogating Met cell membrane half-life 53. BxPC3 cells showed that this receptor has a cell-membrane half-life of approximately 7h (**Fig. 3B**). Additionally, we observed that Met decreases at the cell membrane after 3h of incubation with 30 μg/mL of onartuzumab. A decrease in cell membrane Met is observed even after 6h of incubation, suggesting that the receptor has yet to cycle back to the membrane within this timeframe. Because onartuzumab binds to the extracellular domain of Met, we hypothesized that onartuzumab administration in a fractionated regimen (in the period of time where Met is being recycled back to the membrane) will increase its membrane targeting efficiency (**Figure 3B**). Indeed, a fractionated dose of onartuzumab (3 × 10 μg/mL) as illustrated in **Figure 3C** allowed Met recycling to the plasma membrane after a total incubation time of 6h (**Figure 3D**). Additionally, the amount of radiolabeled onartuzumab associated to cell membrane was higher in BxPC3 cells incubated with a fractionated dose than in cells incubated with a single dose (**Figure 3E**). We observed comparable results in the other PDAC cell lines (**Figure S4**).

Premised on our *in vitro* findings, we expected that a fractionated therapeutic protocol would allow for improved PDAC therapy using onartuzumab radiolabeled with the therapeutic radionuclide lutetium-177 (Lu-177). This hypothesis was supported by pilot *in vivo* therapy study in subcutaneous BxPC3 tumors performed with [^177^Lu]Lu-DTPA-onartuzumab RLT agent, which demonstrated a modest, but statistically significant decrease in tumor growth and overall survival benefit in the cohort of mice bearing BxPC3 tumors treated with three fractions (1.48 MBq(40μCi)/6.7μg × 3 administered at 3 day intervals) versus a single fraction (4.44 MBq(120μCi) /20μg × 1) (data not shown). These promising results prompted a more rigorous evaluation with subsequent experiments.

### Fractionated, Met-directed therapy inhibits pancreatic cancer growth and improves overall survival

**Figure 4:**
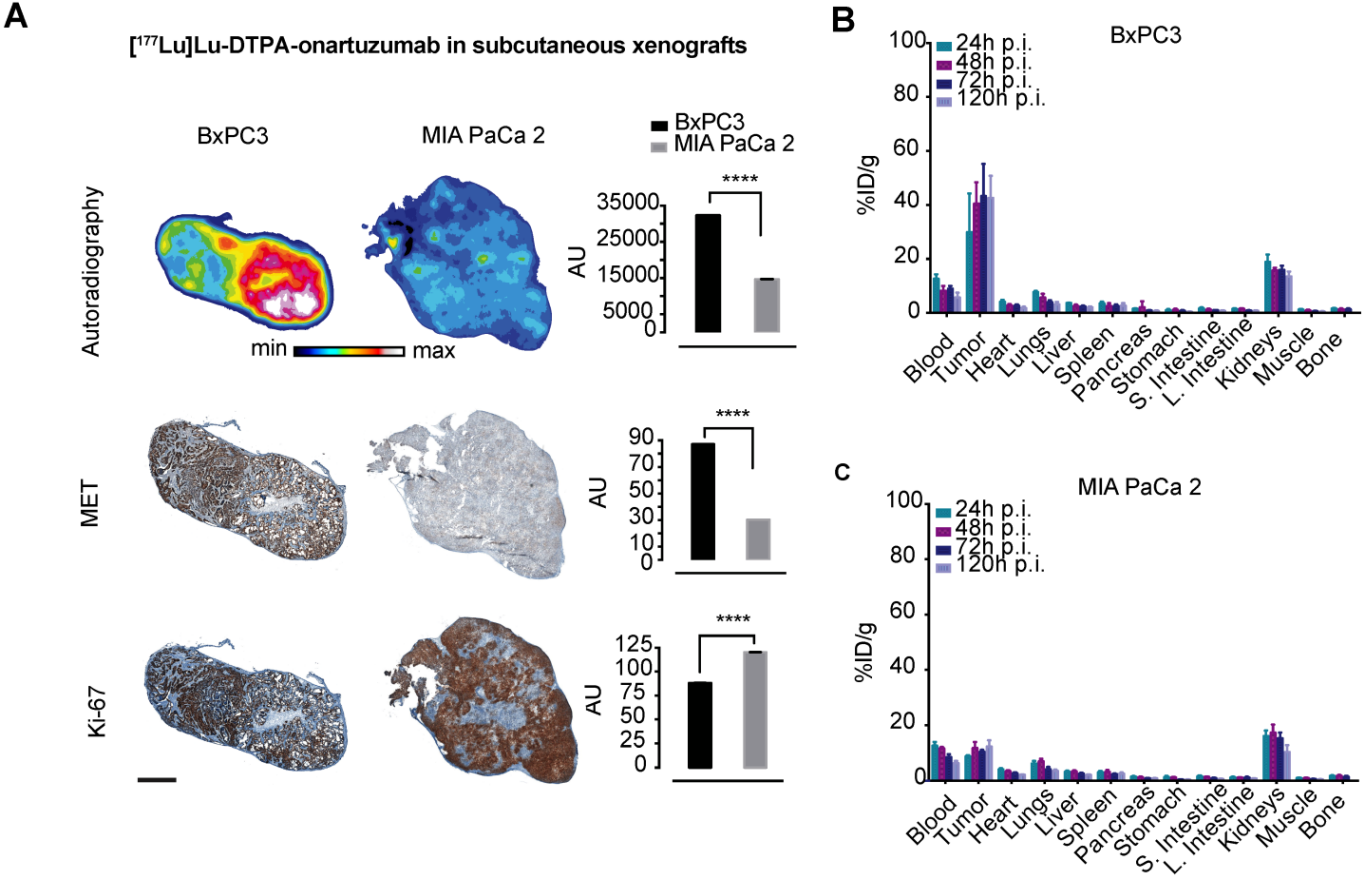
** [^177^Lu]Lu-DTPA-onartuzumab accumulates in tumors expressing Met *in vivo*. A.** Representative autoradiographs, immunohistochemical staining for Met and Ki-67 of BxPC3 and MIA PaCa-2 tumors obtained at 120h post-injection (p.i.) of [^177^Lu]Lu-DTPA-onartuzumab (scale bar: 2000 μm). **B.** ex-vivo tissue biodistribution at 24, 48, 72, and 120h p.i. of [^177^Lu]Lu-DTPA-onartuzumab in animals engrafted with BxPC3 cells, and **C.** MIA PaCa-2 cells**.** AU=arbitrary units is a ratio determined by measuring total of pixels positive for given stain versus the total pixels contained within the tumor. Error bars denote standard deviation (± s.d) of measurement within the representative tumor section, p<0.05 = *, p<0.01= **, p<0.0005 = ***, p<0.0001 = ****, NS: not significant, p >0.05).

Cherenkov imaging, that is, detection of the naturally occurring luminescence of certain radioisotopes, confirmed significantly higher localization of [^177^Lu]Lu-DTPA-onartuzumab in animals bearing BxPC3 tumors compared with those bearing MIA PaCa-2 tumors (**Figure S5**). Autoradiographic evaluation of tumors 120h following injection with [^177^Lu]Lu-DTPA-onartuzumab demonstrated significantly higher activity in BxPC3 tumors, correlating with higher Met expression, and lower Ki-67 staining when compared with MIA PaCa-2 tumors, suggesting proliferative changes resulting from treatment with RLT (**Figure 4A**). Quantitative biodistribution showed rapid accumulation and persistence up to 120h post injection of the construct (**Figure 4B**, **C** and **Table 2**).

**Figure 5:**
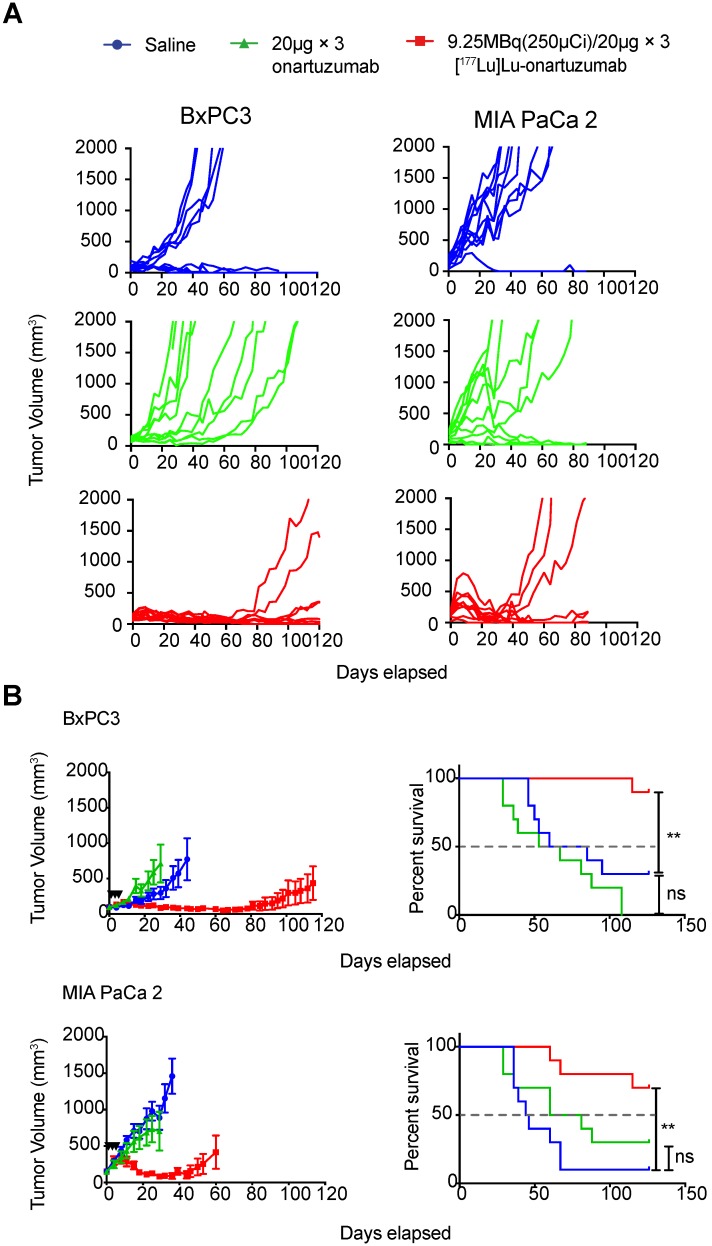
** Targeting of cell-membrane Met with [^177^Lu]Lu-DTPA-onartuzumab results in significant tumor growth delay and overall survival improvement *in vivo*. A.** Spider plots of tumor volumes for BxPC3(n=10 per group) and MIA PaCa-2 (n=10 per group) bearing mice treated with [^177^Lu]Lu-DTPA-onartuzumab 9.25MBq (250µCi)/20µg × 3, every 3 days denoted by black arrowheads) compared with unmodified onartuzumab (20µg × 3, every three days), and saline controls. **B.** MIA PaCa-2 and BxPC3 tumor-engrafted animals demonstrate significant tumor growth delay and improved overall survival on Mantel-Cox log-rank analysis when treated Error bars denote standard error of the mean (± s.e.m), p<0.05 = *, p<0.01= **, p<0.0005 = ***, p<0.0001 = ****, NS: not significant, p >0.05.

Next, we considered whether exceeding the previously determined mass limit of < 25 µg onartuzumab could allow us to localize higher therapeutic activity to tumors, especially given that *in vitro* data suggested that Met recycled back to the cell-membrane after a fractionated regimen of onartuzumab without blocking target engagement. Authors balanced the desire to fractionate doses whilst accounting for radioactive decay of therapeutic Lu-177, and decided to administer three doses of 9.25MBq (250 µCi)/20µg every 72h. Animals engrafted with BxPC3 tumors that were treated with 9.25 MBq(250 µCi)/20µg × 3 fractions exhibited a dramatic tumor growth delay and overall survival benefit as demonstrated in the spider plots of individual tumor growths (**Figure 5A**) as well as the combined mean (**Figure 5B**). Although less dramatic, we were surprised to observe a tumor growth delay and survival benefit in animals bearing MIA PaCa-2 tumors, suggesting that even with relatively low membrane-target availability (**Figure 2B**), if given at sufficient doses and schedule, RLT can have profound therapeutic effect. Notably, we were unable to control for enhanced permeability and retention (EPR) effect with a non-specific one-armed antibody because it was unavailable at the time studies were conducted.

## Discussion

While copy number increases (i.e. amplification) or activating mutations of Met are well-described predictors of response to Met-targeted kinase inhibitors, over-expression and epigenetic alterations might also be important in select cases 54. Reports of a patient with KRAS mutant renal cell carcinoma showed a dramatic response to trametinib (MEK inhibitor) initially, and was found to have Met over-expression upon relapse, which was sensitive to treatment with crizotinib, an ALK and Met inhibitor 55. In KRAS-mutant non-small cell lung cancer (NSCLC) treated with trametinib, Manchado *et al.* demonstrated a compensatory increase in fibroblast growth factor receptor 1 (FGFR1) causing adaptive drug resistance, which when abrogated genetically or pharmacologically, resulted in apparent synthetic lethality and dramatic therapeutic response *in vitro* and *in vivo*. Though not as extensively investigated as KRAS-mutant NSCLC models, promising results of combined trametinib/ponatinib therapy were reported in KRAS-mutant organoid-derived murine model of PDAC as well, suggesting a role for multidrug therapy 8. Interestingly, while Met expression increases were noted in NSCLC cell lines following treatment with trametinib, authors reported lack of response to existing Met TKIs, consistent with a more complex mechanism. This lack of response could result from an interplay between several RTK classes, as was demonstrated by Sanchez-Vega *et al.,* who found that Met, HER2, and EGFR receptor dynamics affected response to pan-EGFR TKI, afatinib 11. Others have demonstrated that abrogating KRAS activation via CRISPR/Cas9 has a limited effect on oncogenicity of PDAC lines due to compensatory signaling changes, further complicating therapeutic strategies for presumed KRAS driven tumors 7.

Our findings confirm that the over-expression of Met is a poor predictor for response to Met-directed TKIs in human PDAC lines, even in the presence of a MEK inhibitor to mitigate KRAS-pathway activation. These findings are in contrast to previous reports suggesting a therapeutic efficacy of Met inhibition in KRAS mutant tumors, albeit with TKIs that are more promiscuous than capmatinib 17. Accordingly, harnessing a cell signaling-agnostic approach may prove useful for diagnosis and therapy.

Compounded with data demonstrating that PDAC cells respond only modestly to small molecule inhibition alone or in combination with other small molecule inhibitors, we were surprised to observe a significant and durable local response as well as a survival benefit by delivering cytotoxic radionuclides to tumors expressing both high and relatively low surface Met in our animal studies. Of equal importance was the ability of our imaging probe, [^89^Zr]Zr-DFO-onartuzumab, to predict the therapeutic response, that is, tumors with higher SUVmax demonstrated a more durable response to [^177^Lu]Lu-DTPA-onartuzumab. While the durability of response was more pronounced in the animals treated with [^177^Lu]Lu-DTPA-onartuzumab harboring BxPC3 tumors, which have high Met expression, animals engrafted with MIA PaCa-2 cells, which have low expression of Met, also exhibited a benefit when treated with our RLT agent. The tumor growth delay and overall survival benefit observed in MIA PaCa-2 cells after administration of a fractionated dose likely reflects a combination of the enhanced permeability and retention effect, non-zero Met expression levels, receptor recycling, and higher radiosensitivity of MIA PaCa-2 compared with BxPC3. Notably, clonogenic assays performed by Souchek *et al.* and Milanovic *et al.* determined that MIA PaCa-2 is an order of magnitude more radiosensitive compared to BxPC3 (surviving fractions of 0.01 and 0.1, respectively, assessed 72 h after 7Gy under similar conditions, for example) 56, 57. Authors also note that, despite randomizing animals to groups such that the mean tumor volumes were comparable prior to treatment administration (BxPC3 = 100±40.5 mm^3^ and MIAPaCa-2 = 150±68.4 mm^3^), four animals in the BxPC3 and one animal in the MIA PaCa-2 saline treatment groups demonstrated tumor regression, which represents a limitation of the models used in our study. Since we could not definitively confirm that animals with tumor regression in the treatment arms were primarily due to our treatment, authors decided to include these animals in the final analyses. That said, inclusion of these animals would bias the results against the efficacy of our RLT and the fact that we observed significant local control and survival benefits despite this handicap speaks to the promise of RLT as a modality.

Although Met is overexpressed in pancreatic cancer cells, the cell membrane presence of Met is highly dynamic, which contributes to its short membrane half-life 58. Furthermore, an increase in the internalization and degradation and/or inhibition of Met trafficking from the intracellular compartment to the plasma membrane may also affect its availability 59. We have recently demonstrated that membrane dynamics of receptors (e.g. HER family members) need to be considered when selecting patients for antibody-based imaging and therapeutic approaches 53. Given that the ability of radiolabeled onartuzumab to target cancer cells depends on membrane available Met, we looked to assess whether fractionated administration would alter the membrane availability of Met. Here, we demonstrate that membrane Met increases when radiolabeled onartuzumab was given in a fractionated approach and suggests a possible rationale for this dosing schema. An open question is the extent to which fractionated dosing of systemic radiotherapy improves therapeutic outcome. While our *in vitro* studies demonstrate a relatively short membrane half-life of Met (<8h), and increased membrane Met when incubated with a fractionated dose of the radioligand, the kinetics of this cycling are more challenging to ascertain *in vivo*. The fact that we observed a local response and overall survival benefit, suggests that our treatment dose and schedule were within a favorable time-frame to engage Met on tumor cell membranes. It is possible that the relatively rapid recycling of the receptor may allow for more available binding for subsequent boluses of RLT, though to what extent the receptor dynamics *in vitro* mirror those *in vivo* is unclear. Studies to further characterize this effect as well as defining the mechanisms of RLT resistance are ongoing.

Clearly, deconvoluting the network of signaling cascades involved in oncogenesis of pancreatic and other cancers is crucial to our understanding of the biology that underpins these diseases. Further, it informs the development of targeted small molecule drugs and rational combinations therein. Nevertheless, it is important to highlight that our agent can achieve tumor control and survival benefit *in vivo* despite Met not being a key driver in this disease by exploiting its overrepresentation on tumor over normal tissues. Moreover, while over-expression of Met does not alone predict for response to anti-Met TKIs, immunoPET can assess *in vivo* expression, serving as an important companion imaging biomarker that predicts for therapeutic response to radioligand therapy. This strategy can be applied to not only other Met-expressing cancers specifically, but also may present a blueprint applicable to other differentially overexpressed surface tumor markers. In fact, two open Phase I trials listed on clinicaltrials.gov aim to do just that using, HuMab5B1, a monoclonal antibody specific to CA19.9, a well-known serum and tissue biomarker for pancreatic cancer: NCT02687230 (Zr-89 labeled HuMab-5B1, a.k.a. MVT-2163) and NCT03118349 (Lu-177 labeled HuMab-5B1, a.k.a. MVT-1075). While both trials include other CA19.9-positive malignancies, results will provide insights on how immunoPET could inform RLT more broadly.

## Supplementary Material

Supplementary methods, results, and figures.

## Figures and Tables

**Table 1:** Values for cytotoxic concentration (IC_50_, μM) of kinase inhibitors on human pancreatic cancer cell lines, BxPC3, Capan 2, Suit 2 and MIA PaCa-2.

Cell line	Drug	IC50_24h_ μM (95% CI)	IC50_48h_ μM (95% CI)	IC50_72h_ μM (95% CI)
BxPC3	crizotinib	15.60 (10.03 - 25.28)	0.005 (indeterminate)*	7.01 (4.67 - 10.29)
	cabozantinib	18.99 (13.95 - 27.12)	17.33 (12.08 - 27.05)	13.76 (9.97 - 19.35)
	Capmatinib (INC280)	indeterminate	indeterminate	indeterminate
Capan 2	crizotinib	29.25 (26.22 - 32.26)	19.37 (? - 23.31)	12.08 (9.60 - 14.80)
	cabozantinib	indeterminate	38.20 (?- 66.08)	40.5 (29.90 - 93.93)
	capmatinib (INC280)	indeterminate	indeterminate	indeterminate
Suit 2	crizotinib	18.97 (? - 24.03)	13.44 (8.438 - 19.89)	11.46 (9.868 - 13.27)
	cabozantinib	25.41 (19.81 - 36.70)	36.66 (26.00 - 63.36)	20.81 (14.10 - 36.80)
	capmatinib (INC280)	indeterminate	indeterminate	indeterminate
MIA PaCa-2	crizotinib	indeterminate	10.14 (5.787 - 16.21)	6.41 (4.177 - 9.254)
	cabozantinib	indeterminate	indeterminate	14.30 (10.30 - 20.65)
	capmatinib (INC280)	indeterminate	indeterminate	indeterminate

IC50: concentration of kinase inhibitors required to reduce cell viability by 50% as compared to the control cells. CI: confidence interval. Estimated using non-linear fit variable slope four parameters, bottom=0, with Graphpad Prism. ? = not available due to poor curve fit. *no range reported by software, suggesting poor fit and unreliable IC50 value. “?” represents no reportable value by software, suggesting poor fit and unreliable IC50 estimate.

**Table 2:** *Ex vivo* analysis of [^177^Lu]DTPA-onartuzumab uptake in PDACs MIA PaCa2 and BxPC3 xenograft tumors as percent injected dose per gram tumor (%ID/g).

	MIA PaCa-2 (%ID/g)	BxPC3 (%ID/g)	
**24h p.i.**	8.85±0.4	29.9±14	p=0.03
**48h p.i.**	11.8±2.1	40.5±7.9	p=0.0008
**72h p.i.**	10.5±0.8	43.3±12	p=0.003
**120h p.i.**	12.3±2.2	42.6±8.2	p=0.004

p.i.:post intravenous injection, PDAC: pancreatic ductal adenocarcinoma. Groups of 4-5 animals. p-value determined via two tailed t-test.

## References

[1] Zhan HX, Xu JW, Wu D, Wu ZY, Wang L, Hu SY (2017). Neoadjuvant therapy in pancreatic cancer: a systematic review and meta-analysis of prospective studies. Cancer Med.

[2] Balaban EP, Mangu PB, Khorana AA, Shah MA, Mukherjee S, Crane CH (2016). Locally Advanced, Unresectable Pancreatic Cancer: American Society of Clinical Oncology Clinical Practice Guideline. J Clin Oncol.

[3] Conroy T, Hammel P, Hebbar M, Abdelghani MB, Wei AC-c, Raoul J-L (2018). Unicancer GI PRODIGE 24/CCTG PA.6 trial: A multicenter international randomized phase III trial of adjuvant mFOLFIRINOX versus gemcitabine (gem) in patients with resected pancreatic ductal adenocarcinomas. Journal of Clinical Oncology.

[4] Hruban RH, Goggins M, Kern SE (1999). Molecular genetics and related developments in pancreatic cancer. Curr Opin Gastroenterol.

[5] Eser S, Schnieke A, Schneider G, Saur D (2014). Oncogenic KRAS signalling in pancreatic cancer. Br J Cancer.

[6] Cox AD, Fesik SW, Kimmelman AC, Luo J, Der CJ (2014). Drugging the undruggable RAS: Mission possible?. Nat Rev Drug Discov.

[7] Muzumdar MD, Chen PY, Dorans KJ, Chung KM, Bhutkar A, Hong E (2017). Survival of pancreatic cancer cells lacking KRAS function. Nat Commun.

[8] Manchado E, Weissmueller S, Morris JPt, Chen CC, Wullenkord R, Lujambio A (2016). A combinatorial strategy for treating KRAS-mutant lung cancer. Nature.

[9] Zhen DB, Griffith KA, Ruch JM, Camphausen K, Savage JE, Kim EJ (2016). A phase I trial of cabozantinib and gemcitabine in advanced pancreatic cancer. Invest New Drugs.

[10] Prasad V, McCabe C, Mailankody S (2018). Low-value approvals and high prices might incentivize ineffective drug development. Nat Rev Clin Oncol.

[11] Sanchez-Vega F, Hechtman JF, Castel P, Ku GY, Tuvy Y, Won H (2019). EGFR and MET Amplifications Determine Response to HER2 Inhibition in ERBB2-Amplified Esophagogastric Cancer. Cancer Discovery.

[12] Bensch F, van der Veen EL, Lub-de Hooge MN, Jorritsma-Smit A, Boellaard R, Kok IC (2018). (89)Zr-atezolizumab imaging as a non-invasive approach to assess clinical response to PD-L1 blockade in cancer. Nat Med.

[13] The Human Protein Atlas. Accessed Aug 2019.

[14] Bottaro DP, Rubin JS, Faletto DL, Chan AM, Kmiecik TE, Vande Woude GF (1991). Identification of the hepatocyte growth factor receptor as the c-met proto-oncogene product. Science.

[15] Yu J, Ohuchida K, Mizumoto K, Ishikawa N, Ogura Y, Yamada D (2006). Overexpression of c-met in the early stage of pancreatic carcinogenesis; altered expression is not sufficient for progression from chronic pancreatitis to pancreatic cancer. World J Gastroenterol.

[16] Di Renzo MF, Poulsom R, Olivero M, Comoglio PM, Lemoine NR (1995). Expression of the Met/hepatocyte growth factor receptor in human pancreatic cancer. Cancer Res.

[17] Fujita-Sato S, Galeas J, Truitt M, Pitt C, Urisman A, Bandyopadhyay S (2015). Enhanced MET Translation and Signaling Sustains K-Ras-Driven Proliferation under Anchorage-Independent Growth Conditions. Cancer Res.

[18] Eckhardt SG, Lieu C (2019). Is Precision Medicine an Oxymoron?. JAMA Oncology.

[19] Janjigian YY, Viola-Villegas N, Holland JP, Divilov V, Carlin SD, Gomes-DaGama EM (2013). Monitoring Afatinib Treatment in HER2-Positive Gastric Cancer with (18)F-FDG and (89)Zr-Trastuzumab PET. J Nucl Med.

[20] Larson SM, Nelp WB (1965). Visualization of the placenta by radioisotope photoscanning using technetium-99m-labeled albumin. Am J Obstet Gynecol.

[21] Larson SM, Carrasquillo JA, McGuffin RW, Krohn KA, Ferens JM, Hill LD (1985). Use of I-131 labeled, murine Fab against a high molecular weight antigen of human melanoma: preliminary experience. Radiology.

[22] Esteban JM, Schlom J, Gansow OA, Atcher RW, Brechbiel MW, Simpson DE (1987). New method for the chelation of indium-111 to monoclonal antibodies: biodistribution and imaging of athymic mice bearing human colon carcinoma xenografts. J Nucl Med.

[23] Brechbiel MW, Gansow OA (1991). Backbone-substituted DTPA ligands for 90Y radioimmunotherapy. Bioconjug Chem.

[24] Strosberg J, El-Haddad G, Wolin E, Hendifar A, Yao J, Chasen B (2017). Phase 3 Trial of (177)Lu-Dotatate for Midgut Neuroendocrine Tumors. N Engl J Med.

[25] Doelen MJvd, Mehra N, Smits M, Oort IMv, Janssen MJR, Haberkorn U (2018). Clinical experience with PSMA-Actinium-225 (Ac-225) radioligand therapy (RLT) in end-stage metastatic castration-resistant prostate cancer (mCRPC) patients. J Clin Oncol.

[26] Hofman MS, Violet J, Hicks RJ, Ferdinandus J, Thang SP, Akhurst T (2018). [177Lu]-PSMA-617 radionuclide treatment in patients with metastatic castration-resistant prostate cancer (LuPSMA trial): a single-centre, single-arm, phase 2 study. Lancet Oncol.

[27] Kessel K, Seifert R, Schafers M, Weckesser M, Schlack K, Boegemann M (2019). Second line chemotherapy and visceral metastases are associated with poor survival in patients with mCRPC receiving (177)Lu-PSMA-617. Theranostics.

[28] Merchant M, Ma X, Maun HR, Zheng Z, Peng J, Romero M (2013). Monovalent antibody design and mechanism of action of onartuzumab, a MET antagonist with anti-tumor activity as a therapeutic agent. Proc Natl Acad Sci U S A.

[29] Terwisscha van Scheltinga AG, Lub-de Hooge MN, Hinner MJ, Verheijen RB, Allersdorfer A, Hulsmeyer M (2014). *In vivo* visualization of MET tumor expression and anticalin biodistribution with the MET-specific anticalin 89Zr-PRS-110 PET tracer. J Nucl Med.

[30] Jagoda EM, Lang L, Bhadrasetty V, Histed S, Williams M, Kramer-Marek G (2012). Immuno-PET of the hepatocyte growth factor receptor Met using the 1-armed antibody onartuzumab. J Nucl Med.

[31] Jin H, Yang R, Zheng Z, Romero M, Ross J, Bou-Reslan H (2008). MetMAb, the one-armed 5D5 anti-c-Met antibody, inhibits orthotopic pancreatic tumor growth and improves survival. Cancer Res.

[32] Pool M, Terwisscha van Scheltinga AGT, Kol A, Giesen D, de Vries EGE, Lub-de Hooge MN (2017). (89)Zr-Onartuzumab PET imaging of c-MET receptor dynamics. Eur J Nucl Med Mol Imaging.

[33] Fay R, Gut M, Holland JP (2019). Photoradiosynthesis of 68Ga-Labeled HBED-CC-Azepin-MetMAb for Immuno-PET of c-MET Receptors. Bioconjugate Chemistry.

[34] Segara D, Biankin AV, Kench JG, Langusch CC, Dawson AC, Skalicky DA (2005). Expression of HOXB2, a retinoic acid signaling target in pancreatic cancer and pancreatic intraepithelial neoplasia. Clin Cancer Res.

[35] Badea L, Herlea V, Dima SO, Dumitrascu T, Popescu I (2008). Combined gene expression analysis of whole-tissue and microdissected pancreatic ductal adenocarcinoma identifies genes specifically overexpressed in tumor epithelia. Hepatogastroenterology.

[36] Dyrskjot L, Thykjaer T, Kruhoffer M, Jensen JL, Marcussen N, Hamilton-Dutoit S (2003). Identifying distinct classes of bladder carcinoma using microarrays. Nat Genet.

[37] Sanchez-Carbayo M, Socci ND, Lozano J, Saint F, Cordon-Cardo C (2006). Defining molecular profiles of poor outcome in patients with invasive bladder cancer using oligonucleotide microarrays. J Clin Oncol.

[38] Scotto L, Narayan G, Nandula SV, Arias-Pulido H, Subramaniyam S, Schneider A (2008). Identification of copy number gain and overexpressed genes on chromosome arm 20q by an integrative genomic approach in cervical cancer: potential role in progression. Genes Chromosomes Cancer.

[39] Gaedcke J, Grade M, Jung K, Camps J, Jo P, Emons G (2010). Mutated KRAS results in overexpression of DUSP4, a MAP-kinase phosphatase, and SMYD3, a histone methyltransferase, in rectal carcinomas. Genes Chromosomes Cancer.

[40] Hong Y, Downey T, Eu KW, Koh PK, Cheah PY (2010). A 'metastasis-prone' signature for early-stage mismatch-repair proficient sporadic colorectal cancer patients and its implications for possible therapeutics. Clin Exp Metastasis.

[41] Skrzypczak M, Goryca K, Rubel T, Paziewska A, Mikula M, Jarosz D (2010). Modeling oncogenic signaling in colon tumors by multidirectional analyses of microarray data directed for maximization of analytical reliability. PloS one.

[42] Barretina J, Caponigro G, Stransky N, Venkatesan K, Margolin AA, Kim S (2012). The Cancer Cell Line Encyclopedia enables predictive modelling of anticancer drug sensitivity. Nature.

[43] Price EW, Carnazza KE, Carlin SD, Cho A, Edwards KJ, Sevak KK (2017). (89)Zr-DFO-AMG102 Immuno-PET to Determine Local Hepatocyte Growth Factor Protein Levels in Tumors for Enhanced Patient Selection. Journal of nuclear medicine: official publication, Society of Nuclear Medicine.

[44] Escorcia FE, Steckler JM, Abdel-Atti D, Price EW, Carlin SD, Scholz WW (2018). Tumor-Specific Zr-89 Immuno-PET Imaging in a Human Bladder Cancer Model. Mol Imaging Biol.

[45] Houghton JL, Abdel-Atti D, Scholz WW, Lewis JS (2017). Preloading with Unlabeled CA19.9 Targeted Human Monoclonal Antibody Leads to Improved PET Imaging with (89)Zr-5B1. Mol Pharm.

[46] Vosjan MJ, Perk LR, Visser GW, Budde M, Jurek P, Kiefer GE (2010). Conjugation and radiolabeling of monoclonal antibodies with zirconium-89 for PET imaging using the bifunctional chelate p-isothiocyanatobenzyl-desferrioxamine. Nat Protoc.

[47] Holland JP, Divilov V, Bander NH, Smith-Jones PM, Larson SM, Lewis JS (2010). 89Zr-DFO-J591 for immunoPET of prostate-specific membrane antigen expression *in vivo*. J Nucl Med.

[48] Verel I, Visser GWM, Boellaard R, Stigter-van Walsum M, Snow GB, van Dongen GAMS (2003). 89Zr Immuno-PET: Comprehensive Procedures for the Production of 89Zr-Labeled Monoclonal Antibodies. J Nucl Med.

[49] Lindmo T, Boven E, Cuttitta F, Fedorko J, Bunn Jr PA (1984). Determination of the immunoreactive function of radiolabeled monoclonal antibodies by linear extrapolation to binding at infinite antigen excess. J Immunol Methods.

[50] Lindmo T, Bunn Jr PA (1986). Determination of the true immunoreactive fraction of monoclonal antibodies after radiolabeling. Methods Enzymol.

[51] Houghton JL, Zeglis BM, Abdel-Atti D, Sawada R, Scholz WW, Lewis JS (2016). Pretargeted Immuno-PET of Pancreatic Cancer: Overcoming Circulating Antigen and Internalized Antibody to Reduce Radiation Doses. J Nucl Med.

[52] Schindelin J, Arganda-Carreras I, Frise E, Kaynig V, Longair M, Pietzsch T (2012). Fiji: an open-source platform for biological-image analysis. Nat Methods.

[53] Pereira PMR, Sharma SK, Carter LM, Edwards KJ, Pourat J, Ragupathi A (2018). Caveolin-1 mediates cellular distribution of HER2 and affects trastuzumab binding and therapeutic efficacy. Nat Commun.

[54] Comoglio PM, Trusolino L, Boccaccio C (2018). Known and novel roles of the MET oncogene in cancer: a coherent approach to targeted therapy. Nat Rev Cancer.

[55] Yao Z, Yaeger R, Rodrik-Outmezguine VS, Tao A, Torres NM, Chang MT (2017). Tumours with class 3 BRAF mutants are sensitive to the inhibition of activated RAS. Nature.

[56] Souchek JJ, Baine MJ, Lin C, Rachagani S, Gupta S, Kaur S (2014). Unbiased analysis of pancreatic cancer radiation resistance reveals cholesterol biosynthesis as a novel target for radiosensitisation. Br J Cancer.

[57] Milanovic D, Firat E, Grosu AL, Niedermann G (2013). Increased radiosensitivity and radiothermosensitivity of human pancreatic MIA PaCa-2 and U251 glioblastoma cell lines treated with the novel Hsp90 inhibitor NVP-HSP990. Radiat Oncol.

[58] Coleman DT, Gray AL, Kridel SJ, Cardelli JA (2016). Palmitoylation regulates the intracellular trafficking and stability of c-Met. Oncotarget.

[59] Ancot F, Leroy C, Muharram G, Lefebvre J, Vicogne J, Lemiere A (2012). Shedding-generated Met receptor fragments can be routed to either the proteasomal or the lysosomal degradation pathway. Traffic.

