# Supplementary material for: ImmunoPET Predicts Response to Met-targeted Radioligand Therapy in Models of Pancreatic Cancer Resistant to Met Kinase Inhibitors
Source: Theranostics. 2020 Jan 1;10(1):151–65. doi: 10.7150/thno.37098 (PMC6929627; doi:10.7150/thno.37098)
Supplement: Supplementary file 1 — Supplementary methods, results, and figures. [file thnov10p0151s1.pdf]

Title : ImmunoPET Predicts Response to Met-targeted Radioligand Therapy in Models of Pancreatic Cancer Resistant to Met Kinase Inhibitors

Freddy E. Escorcia,[1,2,3] Jacob L. Houghton,[4] Dalya Abdel-Atti,[1] Patricia R. Pereira,[1] Andrew Cho, [1,5,6] Nicholas T. Gutsche,[3] Kwamena E. Baidoo,[3] Jason S. Lewis[1,7,8]

[1]Department of Radiology, Memorial Sloan Kettering Cancer Center, 1275 York Avenue, New York, New York, 10065, USA

[2]Department of Radiation Oncology, Memorial Sloan Kettering Cancer Center, 1275 York Avenue, New York, New York, 10065, USA

[3]Molecular Imaging Program, Center for Cancer Research, National Cancer Institute, National Institutes of Health, Bethesda, MD 20814, USA

[4]Department of Radiology and Radiological Sciences, Vanderbilt University Institute of Imaging Science, Nashville, TN 37232, USA

[5]Department of Biochemistry & Structural Biology, Weill Cornell Graduate School, New York City, New York 10065, USA

[6]Weill Cornell/Rockefeller/Sloan Kettering Tri-Institutional MD-PhD Program, New York City, New York 10065, USA

[7]Program in Molecular Pharmacology, Memorial Sloan Kettering Cancer Center, 1275 York Avenue, New York, New York, 10065, USA

[8]Weill Cornell Medical College, 1300 York Ave, New York, New York, 10065, USA

Corresponding author: Freddy E. Escorcia, freddy.escorcia@nih.gov, 240.858.3062

Running title: ImmunoPET Predicts Response to Met-targeting RLT in Pancreatic Cancer

Supplemental Information:

Methods:

Assessing PDAC cell survival in presence of kinase inhibition

BxPC3, Capan 2, Suit 2, and MIA PaCa-2 cell lines were plated into 96-well plates (1×10^3 per well) allowed to attach overnight, incubated with varying concentrations (0-80μM) of kinase inhibitors with known activity to Met (crizotinib, cabozantnib, and capmatinib (INC280), dissolved in DMSO) and viability was assayed with Cell Titer-Glo (Promega, Madison, WI) using manufacturer methods and assayed following after incubation with drugs for 0, 24, 48, and 72h at 37°C. Notably, at 48h, cells were washed and drug containing media was added to control for possible break down of drug over time. Wells containing equivalent DMSO volumes were also assessed to control for DMSO effects.

Antibody mass effect on Biodistribution of [^{89}Zr]Zr-DFO-onartuzumab

Biodistribution at studies of [^{89}Zr]Zr-DFO-onartuzumab were performed with varied mass (10, 25, 50, and 100µg with n=5 per group) of the antibody to gauge the optimal mass needed to administer before saturating available ligands at the tumor and, therefore, decreasing the resulting %ID/g at the tumor and degrading signal-to-noise. Organs were harvested 48h following injection of constructs.

Cerenkov imaging

Prior to sacrifice, mice (n=4-5) were imaged via bioluminescence with the IVIS Spectrum In Vivo Imaging System (Perkin Elmer, Waltham, MA) to assess qualitative localization of [^{177}Lu]Lu-DTPA-onartuzumab *in vivo*.

Bioconjugate evaluation

Mass spectrometry. Onartuzumab-DFO and onartuzumab-DTPA conjugates were compared with unmodified onartuzumab via mass spectrometry to determine the number of moieties covalently bound to onartuzumab. Using molecular weights of 753 g/mol for DFO and 593 g/mol for DTPA MW, number of DFO or DTPA moieties per onartuzumab could be estimated. Briefly, proteins were analyzed on an Exactive Plus EMR Orbitrap system (Thermo Scientific). 1 μg of protein was loaded onto a 4 μM bead size MAbPac RP column (3 x 50 mm, Thermo Scientific) utilizing a Vanquish UHPLC (Thermo Scientific) connected to an Exactive Plus EMR mass spectrometer. The proteins were eluted with a 2 to 100% gradient of 80% Acetonitrile with 0.1% Formic acid over 8 minutes with a flow rate of 0.5 mL/min. The EMR was operated with three sequential orbitrap scan methods that each acquired MS1 at 8,750 resolution with a maximum injection time of 100 ms and an AGC target of 3e6. Scans looked at the range of 600-10000 m/z. The first segment included no additional solvation energy, and had 3 summed microscans, the second segment used 30 eV additional solvation energy and 5 microscans and the third microscan with 50 eV additional solvation energy used 10 microscans. All data was processed in BioPharma Finder 2.0 using the respect algorithm with a maximum mass deviation of 20 ppm with sliding windows.

Bio-layer Interferometry. Binding affinity was determined as previously described[1]. Briefly, biotinylated human Met protein (Acro Biosystems, DE) was diluted to 5 μg/mL in assay buffer (1 × PBS with 0.02% Tween-20) in a 96-well plate and loaded onto streptavidin biosensors (FortéBio, Menlo Park, CA). Each onartuzumab, onartuzumab-DFO, and onartuzumab-DTPA were diluted in assay buffer at various concentrations (1.56, 3.125, 6.25, 12.5, 25, and 50nM) and loaded into the 96-well plate in a final volume of 200 μL. Specific and nonspecific binding wells were made for each concentration. The plate was run on an Octet Red96 system (FortéBio) and analyzed with FortéBio Octet Data Analysis software (v.11).

Serum and buffer stability studies. [^{89}Zr]Zr-DFO-onartuzumab sample was incubated in human serum (EMD Millipore, Temecula, CA) or in PBS at 37°C. Samples were run using radio-iTLC with silica-gel impregnated glass-microfiber paper strips (iTLC-SG, Varian, Lake Forest, CA) using mobile phase of aqueous solution of EDTA (50 mM, pH 5.5), and analyzed using an (AR-2000, Bioscan Inc., Washington, DC).

Pharmacokinetic studies. Female athymic nu/nu mice (Charles River Laboratories, Wilmington, MA) were intravenously injected with 50μCi [^{89}Zr]Zr-DFO-onartuzumab and blood was collected via tail nicks at various time points following administration. Counts per minute (CPM) were determined on Wizard^2 automatic gamma counter. Two-phase nonlinear regression decay was used to fit curve using GraphPad Prism Version 8.0, GraphPad software, La Jolla, CA).

Results:

PDAC lines are resistant to Met-selective monotherapy and combined Met- and MEK inhibition.

We first evaluate sensitivity in the KRAS-activated human PDAC cell lines BxPC3 (KRAS wildtype, but functionally active KRAS-pathway *via* BRAF mutation), Capan2,

Suit2, and MIA PaCa-2 [2]. Crizotinib is a TKI with activity to several kinases including ALK, ROS1 and Met (IC_{50}=11 nM cell free). Cabozantinib has activity to VEGFRs, Axl, and Met (IC_{50}=1.3 nM cell free), among others [3-5]. Despite differential expression of Met, PDAC cell lines were all sensitive to crizotinib and cabozantinib, however, all were resistant to the Met-specific inhibitor capmatinib (IC_{50} = 0.13nM cell free) *ab initio*, confirming that other pathways (e.g. KRAS) were contributing to oncogenic potential. When simultaneously treated with trametinib, to abrogate MEK1/2 activity, and capmatinib, no additional viability effects were noted with the addition of capmatinib (**Figure S1D**).

Onartuzumab bioconjugate stoichiometry. Mass spectrometry demonstrated a mass of unmodified onartuzumab to be 99,161 g/mol (**Figure S2A)**. For DFO-modified onartuzumab, the most abundant species (20.3%) contained four DTPA moieties, and the mean number of DTPA moieties per onartuzumab was 4.4. The majority of onartuzumab remained unmodified (60.6%), while the most abundant species of modified onartuzumab contained a single DTPA moiety (32.5%); the next most common species contained two (5.7%). The mean number of DTPA moieties per onartuzumab were 0.44. Given that onartuzumab and derivatives have very long k_{off}, binding affinity estimates are difficulty to estimate accurately, however, bio-layer interferometry showed subnanomolar affinity for all constructs (**Figure S2B**).

Radiochemistry:

Conjugation with either *p*-SCN-Bn-DFO or *p*-SCN-Bn-CHX-A"-DTPA and subsequent radiolabeling with Zr-89 and Lu-177 resulted in specific activities of 9.3±1.0 Ci/g and 13.1±0.4 Ci/g, respectively. Radiochemical yields and purity were >75% and >95%, respectively, for both constructs and Lindmo immunoreactivity were typically >85%.

Serum stability and pharmacokinetics.

Stability of the [^{89}Zr]Zr-DFO-onartuzumab radioconjugate was determined in serum and PBS. We confirmed that the >60% of radioconjugate remained intact at 37°C in serum (**Figure S2C**). The radioconjugate was far less stable in PBS, with <40% remaining intact by 24h (**Figure S2C**). The blood half-life of the [^{89}Zr]Zr-DFO-onartuzumab was estimated to be 3.6 days in mice (**Figure S2D**).

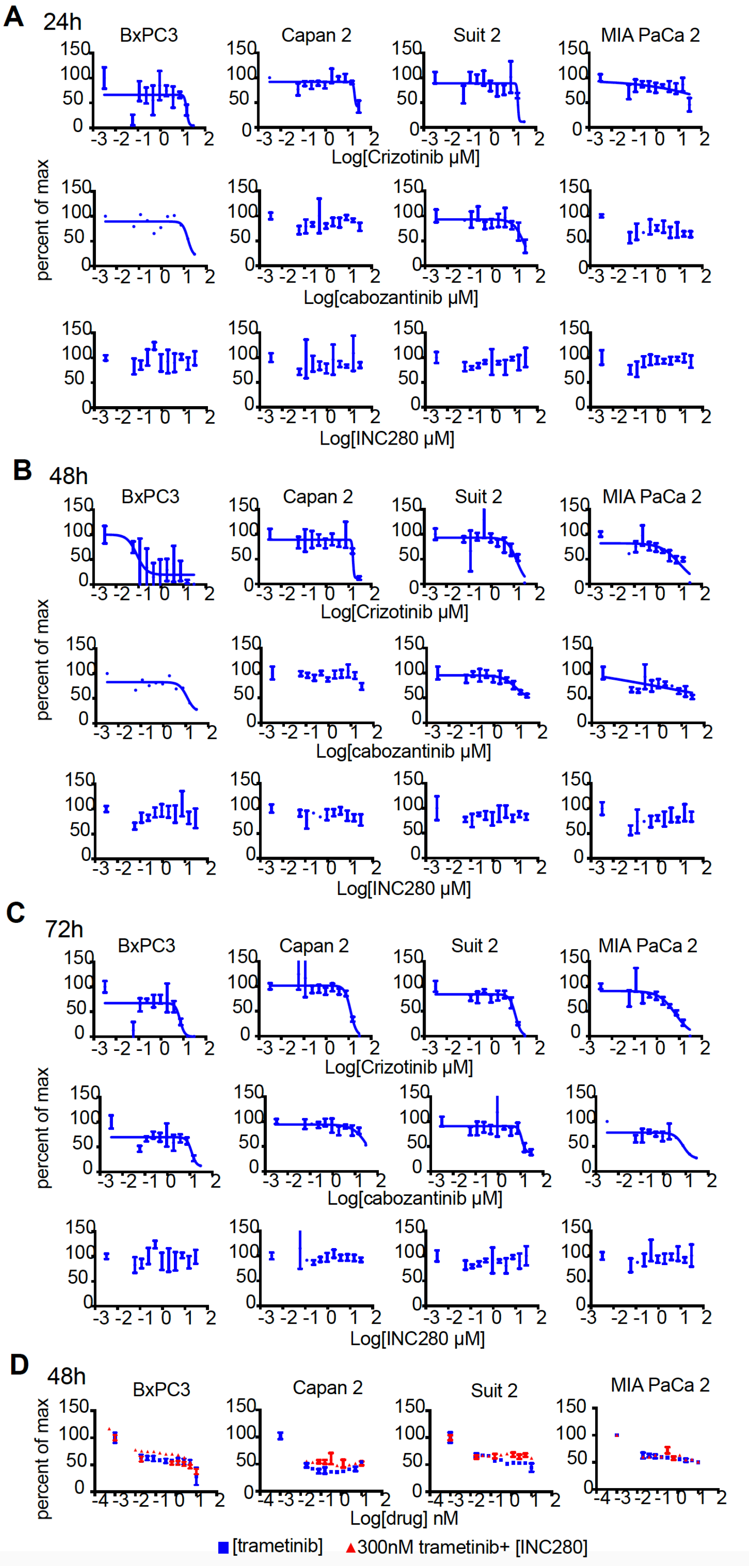
A
24h
BxPC3
Capan 2
Suit 2
MIA PaCa 2
percent of max
Log[Crizotinib μM]
Log[cabozantinib μM]
Log[INC280 μM]
B
48h
BxPC3
Capan 2
Suit 2
MIA PaCa 2
percent of max
Log[Crizotinib μM]
Log[cabozantinib μM]
Log[INC280 μM]
C
72h
BxPC3
Capan 2
Suit 2
MIA PaCa 2
percent of max
Log[Crizotinib μM]
Log[cabozantinib μM]
Log[INC280 μM]
D
48h
BxPC3
Capan 2
Suit 2
MIA PaCa 2
percent of max
Log[drug] nM
[trametinib]
300nM trametinib+ [INC280]

Figure S1: Viability (ATP-quantitation) studies of BxPC3, Capan 2, Suit 2, and MIA PaCa-2 incubated for **A.** 24h **B.** 48h, and **C.** 72h with tyrosine kinase inhibitors with Met activity crizotinib, cabozantinib and capmatinib (INC280). **D**. cell lines incubated with 300mM trametinib and varying concentrations (nM) of capmatinib.

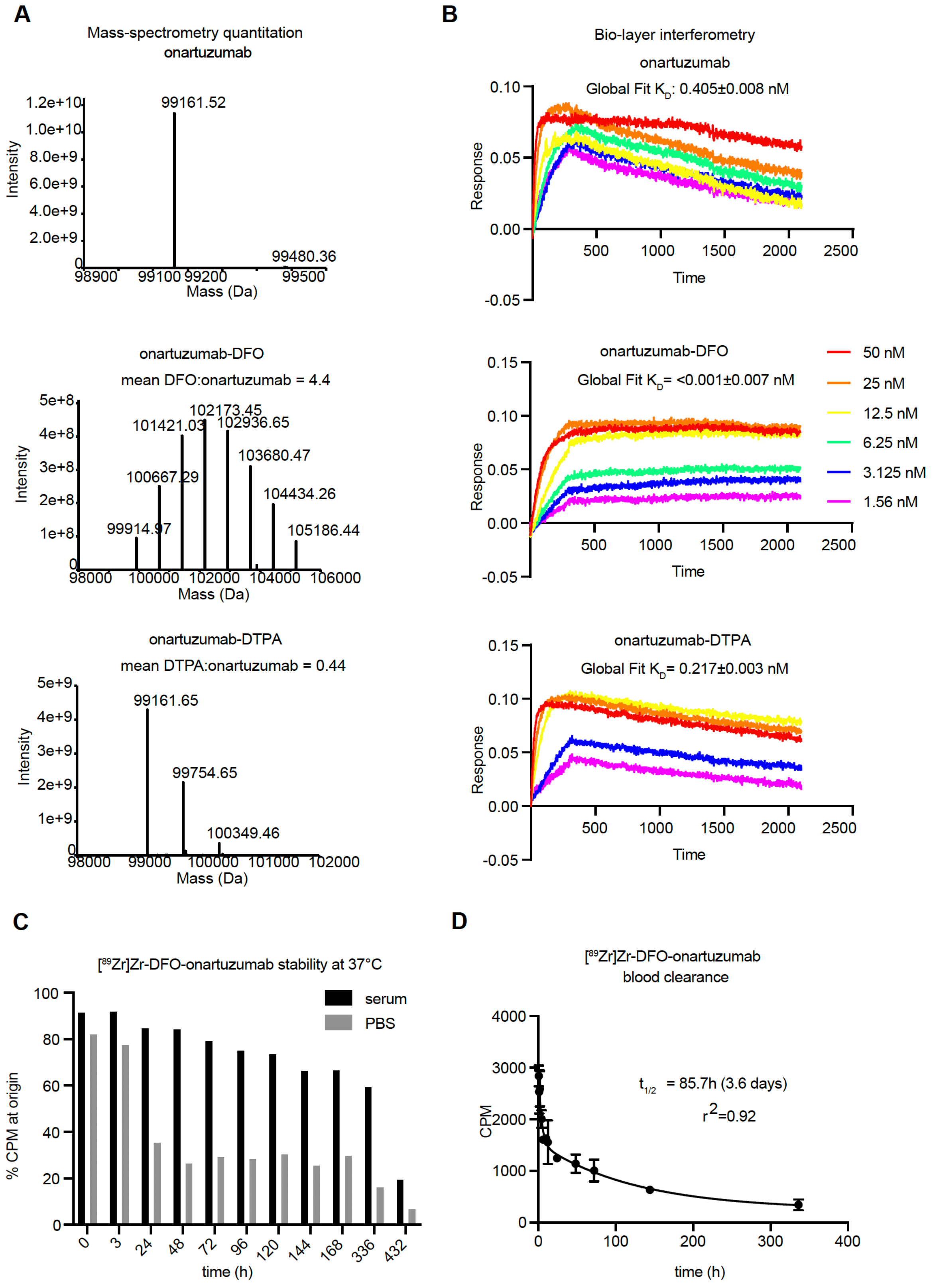
A
Mass-spectrometry quantitation
onartuzumab
Intensity
Mass (Da)
99161.52
99480.36
onartuzumab-DFO
mean DFO:onartuzumab = 4.4
99914.97
100667.29
101421.03
102173.45
102936.65
103680.47
104434.26
105186.44
onartuzumab-DTPA
mean DTPA:onartuzumab = 0.44
99161.65
99754.65
100349.46
B
Bio-layer interferometry
onartuzumab
Global Fit K_D: 0.405±0.008 nM
onartuzumab-DFO
Global Fit K_D= <0.001±0.007 nM
onartuzumab-DTPA
Global Fit K_D= 0.217±0.003 nM
Response
Time
50 nM
25 nM
12.5 nM
6.25 nM
3.125 nM
1.56 nM
C
[89Zr]Zr-DFO-onartuzumab stability at 37°C
serum
PBS
% CPM at origin
time (h)
D
[89Zr]Zr-DFO-onartuzumab
blood clearance
t1/2 = 85.7h (3.6 days)
r2=0.92
CPM
time (h)

Figure S2. **A.** Mass spectra of unmodified onartuzumab, onartuzumab-DFO, and onartuzumab-DTPA confirms bioconjugates exhibited covalent modification with 4.4 DFO and 0.44 DTPA per onartuzumab, respectively. **B.** Bio-layer interferometry curves for onartuzumab, onartuzumab-DFO, and onartuzumab-DTPA with associated dissociation constant estimates, K_D. **C.** [^{89}Zr]Zr-DFO-onartuzumab stability in serum or PBS at 37°C plotted as a percent of total activity remaining at the origin of the iTLC strip. **D.** Blood clearance of diagnostic radioligand in vivo.

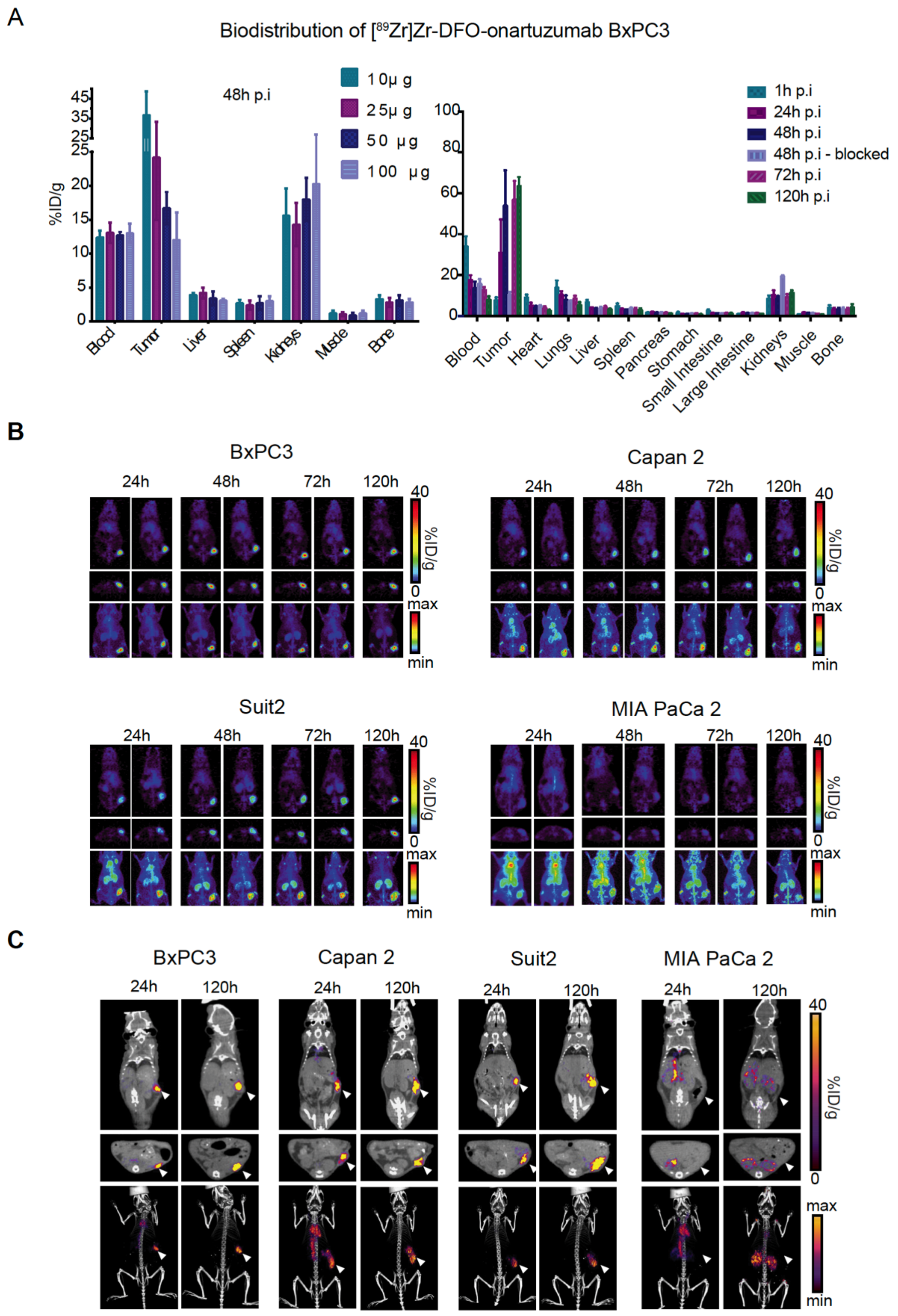

Figure S3. A, left. Quantitative biodistribution of varying mass of [89Zr]Zr-DFO-onartuzumab in BxPC3 engrafted animals to determine maximum mass to be

injected in therapeutic studies before saturation of in vivo target. Significant decrement in tumor %ID/g was noted between 10μg versus 50μg mass of onartuzumab (p=0.0062), while no difference was noted between 10μg and 25μg, suggesting that maximum injected mass below 25μg would be optimal for tumor accumulation. **A, right.** Quantitative biodistribution of [^{89}Zr]Zr-DFO-onartuzumab in mice subcutaneously engrafted with BxPC3 at varying time points post injection (p.i.) denoted as percent injected dose per gram (%ID/g). **B.** Representative coronal, axial slices (top and center), and maximum intensity projection (bottom) PET images of human pancreatic cancer cell lines subcutaneously engrafted into mice evaluated at 24, 48, 72, and 120h post injection. **C.** Representative PET images of human pancreatic cancer cell lines orthotopically engrafted into mice evaluated at 24, and 120h post injection. Error bars denote standard deviation. Error bars denote standard error of the mean (± s.e.m).

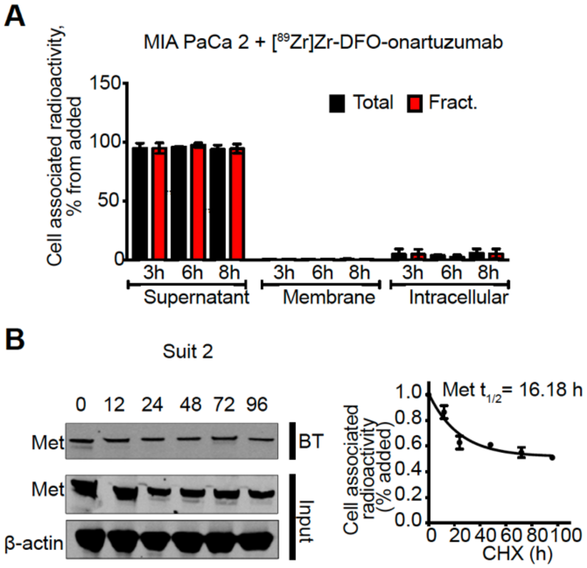

Figure S4: A. MIA PaCa-2 cells treated with fractionated cellular localization of [^{89}Zr]Zr-DFO-onartuzumab in MiaPaCa-2 cells. MiaPaCa-2cells were incubated with a total or a fractionated dose (given every 3h) of [^{89}Zr]Zr-DFO-onartuzumab and radioactivity was measured at 3, 6 and 8 h post treatment in supernatant, membrane and intracellular fractions. **B.** Western blot of biotinylated cell surface–associated Met along with Met input in the total lysates of Suit 2 cells after blocking protein synthesis with 80 μg/mL CHX for 0, 12, 24, 48, 72 and 96h. CHX, cyclohexamide. Half-life of cell surface–associated Met calculated after western blot analysis. Density of western blot bands was quantified by scanning densitometry with ImageJ software. Half-life was calculated as the time required for Met protein decrease to 50% of its initial level.

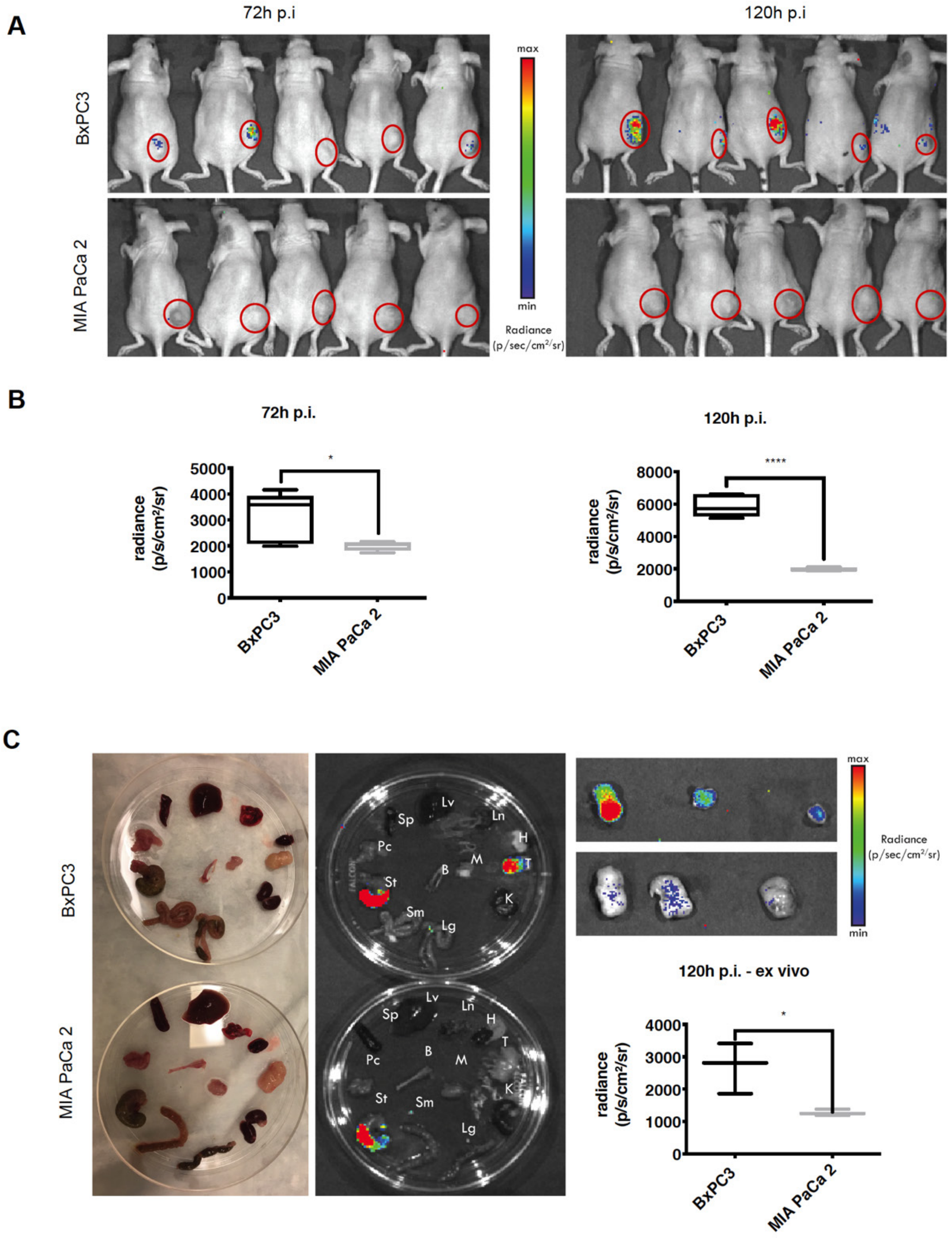

Figure S5. A. Cerenkov imaging of animals engrafted with BxPC3 or MIA PaCa 2 cells 72h and 120h post injection with [^{177}Lu]Lu-DTPA-onartuzumab. **B.** Radiance within region of interest (red circles) was significantly higher in BxPC3 tumors when compared to MIA PaCa 2 tumors. **C.** Organ level Cerenkov imaging and

quantitation confirming higher [^{177}Lu]LuDTPA-onartuzumab-attributable activity in BxPC3 tumors versus MIA PaCa 2 tumors. Signal from stomach attributed to compounds contained in animal feed. Error bars denote standard deviation of the mean (± s.d), p<0.05 = *, p<0.01= **, p<0.0005 = ***, p<0.0001 = ****, NS: not significant, p <0.05. Note: for **c.** the s.d. was instrument-level and not a biological replicate.

Supplemental References

1. Berman RM, Kelada OJ, Gutsche NT, Natarajan R, Swenson RE, Fu Y, et al. In Vitro Performance of Published Glypican 3-Targeting Peptides TJ12P1 and L5 Indicates Lack of Specificity and Potency. Cancer Biother Radiopharm. 2019.
2. Chen SH, Zhang Y, Van Horn RD, Yin T, Buchanan S, Yadav V, et al. Oncogenic BRAF Deletions That Function as Homodimers and Are Sensitive to Inhibition by RAF Dimer Inhibitor LY3009120. Cancer Discov. 2016; 6: 300-15.
3. Liu X, Wang Q, Yang G, Marando C, Koblish HK, Hall LM, et al. A Novel Kinase Inhibitor, INCB28060, Blocks c-MET–Dependent Signaling, Neoplastic Activities, and Cross-Talk with EGFR and HER-3. Clin Cancer Res. 2011; 17: 7127-38.
4. Zou HY, Li Q, Lee JH, Arango ME, McDonnell SR, Yamazaki S, et al. An Orally Available Small-Molecule Inhibitor of c-Met, PF-2341066, Exhibits Cytoreductive Antitumor Efficacy through Antiproliferative and Antiangiogenic Mechanisms. Cancer Res. 2007; 67: 4408-17.
5. Yakes FM, Chen J, Tan J, Yamaguchi K, Shi Y, Yu P, et al. Cabozantinib (XL184), a Novel MET and VEGFR2 Inhibitor, Simultaneously Suppresses Metastasis, Angiogenesis, and Tumor Growth. Mol Cancer Ther. 2011; 10: 2298-308.
